# Fibroblast-specific activation of Rnd3 protects against cardiac remodeling in diabetic cardiomyopathy via suppression of Notch and TGF-β signaling

**DOI:** 10.7150/thno.77043

**Published:** 2022-10-17

**Authors:** Yan Zhang, Yang Cao, Rui Zheng, Zhenyu Xiong, Zhengru Zhu, Fanya Gao, Wanrong Man, Yu Duan, Jie Lin, Xuebin Zhang, Dexi Wu, Mengyuan Jiang, Xiao Zhang, Congye Li, Xiaoming Gu, Yanhong Fan, Dongdong Sun

**Affiliations:** 1 Department of Cardiology, Xijing Hospital, Fourth Military Medical University, Xi'an, China.; 2 Department of Biochemistry and Molecular Biology, Fourth Military Medical University, Xi'an, China.; 3 Department of Otolaryngology, Xijing Hospital, Fourth Military Medical University, Xi'an, China.; 4 Department of General Practice, Xijing Hospital, Fourth Military Medical University, Xi'an, China.; 5 Department of Physiology and Pathophysiology, Fourth Military Medical University, Xi'an, China.

**Keywords:** Cardiac remodeling, Cardiac fibrosis, Rho-GTPase, Cardiac fibroblast, Oxidative stress

## Abstract

**Rationale:** Extracellular matrix (ECM) remodeling, a key pathological feature in diabetic cardiomyopathy (DCM), is triggered by oxidative stress, inflammation, and various metabolic disorders in the heart. Cardiac fibroblasts (CFs) are the primary source of ECM proteins and the ultimate effector cells in ECM remodeling. CFs are turned on and differentiated into myofibroblasts in response to profibrotic signaling. Rnd3 is a small Rho-GTPase involved in the regulation of cell-cycle distribution, cell migration, and cell morphogenesis. Emerging evidence suggests a link between Rnd3 expression and onset of cardiovascular diseases. However, the role of Rnd3 in DCM remains unknown.

**Methods:** Flow cytometry was employed to separate different types of cardiac cells. Type 2 diabetes mellitus was established in Rnd3 fibroblast-specific knockout and transgenic mice. RNA sequencing and chromatin immunoprecipitation assay were used to discern signaling pathways involved.

**Results:** Rnd3 expression was reduced in cardiac tissues of diabetic mice, with CFs being the primary cell type. Fibroblast-specific upregulation of Rnd3 *in vivo* was protective against DCM, whereas Rnd3 downregulation in fibroblasts accentuated cardiac oxidative stress, fibrosis, ventricular remodeling, and dysfunction. Moreover, *in vitro* Rnd3 overexpression significantly attenuated reactive oxygen species production, CF migration and proliferation under high levels of glucose (35 mmol/L) and palmitic acid (500 µmol/L) challenge. Furthermore, RNA sequencing indicated that Notch and TGF-β signaling were significantly suppressed upon Rnd3 overexpression. Mechanistically, Rnd3 regulated Notch and TGF-β signaling by interacting with NICD and ROCK1, respectively. Specifically, glucotoxicity and lipotoxicity control Rnd3 expression by regulating the binding of Nr1H2 and Rnd3 promoter.

**Conclusions:** Our findings provide compelling evidence in that fibroblast-specific activation of Rnd3 protects against cardiac remodeling in DCM, indicating promises of targeting Rnd3 in the treatment of DCM.

## Introduction

The global prevalence of diabetes mellitus was estimated at 10.5% (equivalent to 536.6 million populations) in 2021, and is expected to increase to 12.2% (783.2 million populations) by 2045 1. Diabetes onset triggers a series of cardiovascular complications in patients. The sharp rise in diabetes incidence is deemed an important factor underlying the high incidence of cardiovascular diseases. On the other side of the coin, cardiovascular disease is the leading cause of death in patients with diabetes 2. Diabetic cardiomyopathy (DCM), driven by hyperglycemia, hyperlipidemia, and insulin resistance in the absence of other cardiac risk factors, is characterized by microvascular injury, extracellular matrix (ECM) remodeling, and cardiac dysfunction 3. However, the precise pathogenesis of this condition remains elusive, heavily impacting effective preventive measures for DCM. Thus, a thorough investigation of the therapeutic targets of DCM holds clinical significance.

ECM remodeling, the key pathological process in the incidence and development of DCM 4, is a phenomenon representing altered composition and quantity of extracellular matrix in response to oxidative stress, mechanical stress, and metabolic disorders 5, 6. Cardiac fibroblasts (CFs) form the primary source of ECM proteins and the ultimate effector cells in ECM remodeling. CFs are turned on and are differentiated into myofibroblasts in response to fibrotic signals 7. These profibrotic responses successively trigger collagen production, ECM accumulation, and cardiac remodeling 8. Therefore, CFs are potential cellular targets for anti-ECM-remodeling therapies for DCM.

Rho family GTPase 3 (Rnd3), also known as RhoE, belongs to the Rnd subgroup of the Rho family of small guanosine triphosphate GTP-binding proteins 9. Emerging evidence has suggested a link between Rnd3 expression and hallmarks of cardiovascular disease progression, such as vascular remodeling in hypertension, developmental arrhythmogenesis, and inflammatory responses in myocardial infarction 10-13. In this context, Rnd3 plays an undisputedly key role in the heart, although its role in ventricular remodeling remains unclear in diabetes. In this study, we examined the role of Rnd3 and related pathways in CFs in the face of DCM.

## Methods

### Animals

Animal experiments were performed according to the Guide for the Care and Use of Laboratory Animals, eighth edition (2011), and were approved by the Fourth Military Medical University Ethics Committee on Animal Care (Approval ID: 2,016,1010). Fibroblast-specific knockout mice were generated by crossing Rnd3 flox homozygous (fl/fl) (Rnd3^fl/fl^KO) mice with col1a2-cre mice. Fibroblast-specific transgenic mice were generated by crossing conditional Rosa26 Rnd3 knockin homozygous (lsp/lsp) (Rnd3^lsp/lsp^Tg) mice with col1a2-cre mice. Rnd3^fl/fl^KO and col1a2-cre mice were purchased from Shanghai Model Organisms Center, Inc. (serial number: 2018-W-3735, China). Rnd3^lsp/lsp^Tg mice were purchased from Cyagen Biosciences Inc. (serial number: TOS161121BA2, China). PCR was used to determine genotypes of experimental mice.

Type 2 diabetes mellitus (T2DM) was induced by administering a high-fat diet containing 60% calories from fat (HFD; Trophic Animal Feed High-Tech Co., Nantong, Jiangsu, China) and 30 mg/kg of streptozotocin (STZ; Sigma-Aldrich, St. Louis, MO, USA) 14-16. Briefly, 8-week-old male mice were fed HFD for 24 weeks, and were then intraperitoneally (i.p.) injected with STZ for 7 days after 8 weeks of HFD feeding. Control mice were fed a normal diet containing 10% calories from fat (Trophic Animal Feed High-Tech Co., Nantong, Jiangsu, China) and received same volume of citrate buffer. Blood glucose levels were monitored regularly using a reflectance meter (Accu-Chek, Roche Diagnostics GmbH, Mannheim, Germany).

### Flow cytometry

Cardiac cell suspensions were prepared as described with modifications 17, 18. In brief, mouse hearts were removed, cut into small pieces, and digested with 2 mg/mL collagenase II and 0.5 mg/mL dispase II for 30 min at 37 °C. The mixture was filtered with a 100 μm filter to remove tissue blocks in the cell dissociation solution. The mixture was then centrifuged at 1000 × *g* for 5 min, and was resuspended with PBS. Cardiac cells were sorted using flow cytometry (Beckman CytoFLEX SRT, Brea, CA, USA) into populations of CD45^-^ PDGFR-α^-^ CD31^-^ cardiomyocytes, CD45^-^ PDGFR-α^+^ CFs, CD45^-^ CD31^+^ endothelial cells, and CD45^+^ F4/80^+^ macrophages. Isolated cells used for RNA extraction and q-PCR analysis.

### Cell culture and treatment

CFs were isolated by enzymatic digestion and were cultured in full DMEM medium as previously described 19-21. CFs isolated from male neonatal C57BL/6J mice (1-3 days) were cultured under normal glucose with 10% fetal bovine serum to the second generation before treatment. Then cells were cultured with serum-free medium 24 hours for synchronization, prior to the usage for ROS production, cell proliferation, and migration. Following synchronization and transfection of adenoviruses, cells were cultured with high levels of glucose (HG; 35 mmol/L) and palmitic acid (PA; 500 μmol/L) in DMEM for another 48 hours to assess ROS production. Normal glucose (NG; 5 mmol/L) was used as the control medium. Mannitol at the same concentration was used as osmolarity control (OC; 5.5 mmol/L glucose and 29.5mmol/L mannitol). CFs isolated from adult mice in different control and experimental groups were cultured under normal glucose for 18 hours for further biochemical assessment.

### Upregulation and downregulation of target genes

For *in vitro* gene delivery, the adenoviruses harboring Rnd3 (Ad-Rnd3), Rnd3 shRNA (Ad-shRnd3), Nr1H2 (Ad-Nr1H2) and control vectors for Ad-Rnd3 (Ad-Control), Rnd3 shRNA (Ad-LacZ), Ad-Nr1H2 (Ad-Control) (Hanbio Technology, Shanghai, China) were used to overexpress or knockdown Rnd3 and Nr1H2. The sequences of shRNA for Rnd3 are 5'-CCAGAGACTCTGGACAGTGTCTTAATTCAAGACACTGTCCAGAGTCTCTGGTT-3' and 5'-CAGAGACTCTGGACAGTGTCTTAATCTAAGACACTGTCCAGAGTCTCTGGC-3', the sequences of shRNA for LacZ are 5'-CACCGCTACACAAATCAGCGATTTCGAAA AATCGCTGATTTGTGTAG-3' and 5'-AAAACTACACAAATCAGCGATTTTTCGAAATCG CTGATTTGTGTAGC-3'. CFs were transduced with adenoviruses at various multiplicities of infection (MOIs) in a FBS-free medium after cells had grown to 50-60% confluence. After 8 h of infection, medium was replaced with fresh medium containing 10% FBS, and cells were harvested 48 h to evaluate various signaling pathways. Please refer to the supplementary methods for additional information.

### Histological analysis and immunohistochemistry staining

Mouse cardiac tissues were fixed overnight with 4% paraformaldehyde, embedded in paraffin, and cut into 5 μm slices. Tissue morphology was determined by hematoxylin and eosin staining, and Masson's trichrome staining was performed to determine the cardiac collagen content. Immunohistochemistry staining of cardiac tissues was performed as described previously 22. Briefly, heart sections were incubated with antibodies against α-SMA at 4 °C overnight, with secondary antibodies at 37 °C for 1 hour and detected using 3, 3'-diaminobenzidine in sequence. Finally, the sections were counterstained with hematoxylin before microscopy.

### Detection of ROS

Dihydroethidium (DHE) fluorescence staining (1:500; Sigma-Aldrich) was performed to assess ROS production. ROS level was determined using red fluorescence under a fluorescence microscope (DP74, Olympus Optical, Tokyo, Japan). For CFs, a ROS Assay Kit (Beyotime Biotechnology, Shanghai, China) was used according to the manufacturer's instructions. Briefly, CFs were incubated with a fluorescent probe DCFH-DA (10 μmol/L) in serum-free DMEM at 37 °C for 30 min after indicated treatment, and were then washed three times with serum-free DMEM. Fluorescence was examined by flow cytometry (CytoFLEX S; Beckman Coulter, CA, USA).

### Cell proliferation and migration assay

Cell proliferation was evaluated using an EdU kit (Beyotime Biotechnology) and cell counting kit-8 (CCK8, Beyotime Biotechnology) according to the manufacturer's protocols. CFs were seeded into 6-well or 96-well microplates (Corning, USA) at a density required by the corresponding protocols. Following a 48-hour treatment, CFs were continuously incubated with EDU (10 μmol/L) or 10 μL of CCK‐8 reagent for 2 hours prior to fluorescent detection. Proliferating cells emitted bright red fluorescence under a fluorescence microscope (DP74, Olympus Optical) in Edu assay. The proliferation of cells in CCK8 assay was expressed by the absorbance. The transwell chemotactic assay was used for cell migration measurement. Cells that migrated through the permeable membrane were stained with a crystal violet staining solution. Refer to the supplementary methods for additional information.

### RNA-sequencing

CFs isolated from neonatal mice were cultured for 8 hours with Ad-Rnd3 or a control reagent at the second generation, and then treated with high glucose and palmitic acid medium for 48 hours. Total RNA was extracted from CFs using a TRIzol reagent (Invitrogen, CA, USA). Genes with significant differential expression genes were identified using the criteria |log2FC| ≥1 and p value<0.05. Gene ontology (GO) was used to annotate the function of genes differentially expressed for molecular functions, cellular components, and biological processes. Differentially expressed genes were analyzed using the Kyoto Encyclopedia of Genes and Genomes (KEGG) pathway. All analyses were performed on the integrated cloud platform of Majorbio (https://cloud.majorbio.com/).

### Chromatin immunoprecipitation (ChIP) assay

DNA chromatin was isolated from CFs using a SimpleChIP Plus Sonication Chromatin IP Kit (Cell Signaling Technology), as described previously 23. After the transfection of adenoviruses 48 hours, a Flag antibody was used to immunoprecipitate the protein-DNA complexes, and mouse IgG was used as the control. The primers listed in the supplementary methods were used to detect ChIP DNA using PCR. Refer to the supplementary methods for details.

### RhoA activity assay

The Rho Activation Assay Biochem Kit (Cytoskeleton Inc., COLO, USA) was used for the GTP-RhoA pull-down assay. Proteins (300 µg) were incubated with agarose-conjugated Rhotekin-RBD (50 µg) at 4 °C for 1 h, following which the beads were washed and recovered by centrifugation at 5000 × *g* at 4 °C for 3 min and boiled with 20 µL of Laemmli sample buffer for western blot analysis. Please refer to supplementary methods for details.

### Statistical analysis

Data are expressed as means ± standard deviation of the mean (SD). An unpaired Student's *t*-test and one-way analysis of variance were employed to determine the difference between two-group or multigroup comparisons using GraphPad Prism 8 (GraphPad Software, La Jolla, CA, USA), followed by a Bonferroni post hoc test (when necessary). P values of less than 0.05 were considered statistically significant.

## Results

### Rnd3 expression decreases in cardiac tissues in diabetic mice, and CFs represent the primary cell type

**Figure 1:**
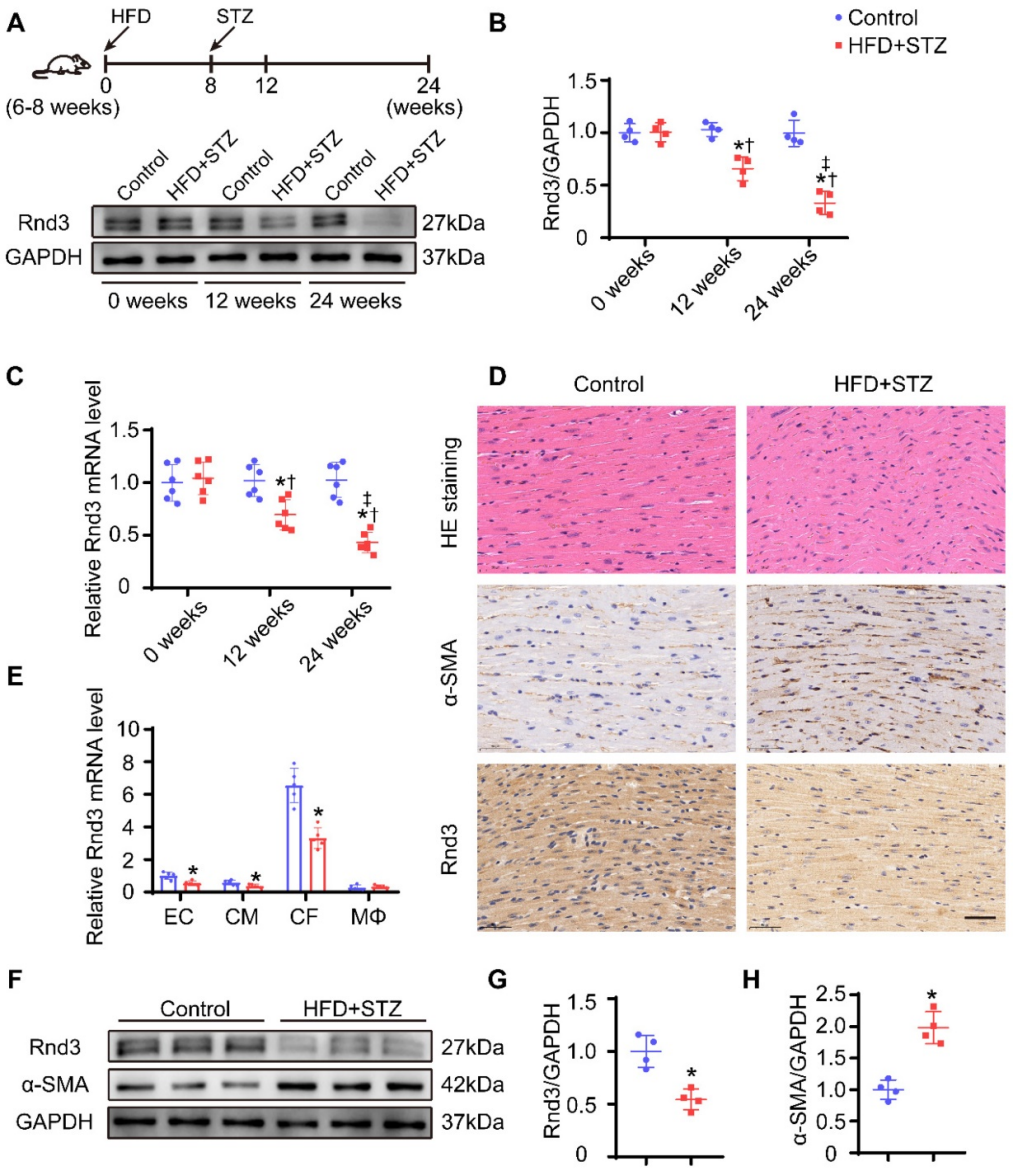
** Rnd3 expression is decreased in cardiac tissues in response to HFD+STZ challenge and cardiac fibroblasts (CFs) are the primary cellular type. (A)** Cardiac Rnd3 protein level evaluated using Western blot from the indicated treatment groups at 0, 12, and 24 weeks. **(B)** Quantitative analysis of Rnd3 protein level from the indicated treatment groups (n = 4). **(C)** Cardiac Rnd3 mRNA level at 0, 12, and 24 weeks evaluated using qPCR (n = 6). *P < 0.05 *vs.* Control; ^†^P < 0.05 *vs.* HFD+STZ at 0 weeks; ^‡^P < 0.05 *vs.* HFD+STZ at 12 weeks. **(D)** Representative HE and immunohistochemical staining of Rnd3 and α-SMA in mouse hearts from the indicated treatment groups at 0 and 24 weeks (n = 4); scale bars represent 40 µm. **(E)** qPCR analysis of Rnd3 expression in endothelial cells (ECs), cardiomyocytes (CMs), CFs, and macrophages (MΦ) at 0 and 24 weeks after HFD+STZ treatment (n = 5). **(F)** Rnd3 and α-SMA expression evaluated using Western blot in CFs at 0 and 24 weeks following the indicated treatments. **(G-H)** Quantitative analysis of Rnd3 and α-SMA protein level after the indicated treatments (n = 4). *P < 0.05 *vs.* Control.

To gain insights into the possible role of Rnd3 in the pathophysiology of DCM, Rnd3 levels were evaluated in cardiac tissues of HFD-STZ-induced diabetic mice. Western blot analysis revealed downregulation of Rnd3 expression in a time-dependent manner in hearts from HFD-STZ treated mice compared with those from age-matched control mice (Figure 1A-B). Meanwhile, qPCR also revealed decreased Rnd3 mRNA expression in hearts from HFD-STZ-treated mice (Figure 1C). Concomitantly, similar findings were noted in paraffin sections. HE staining exhibited hypertrophy and myofibrillar disarray in diabetic hearts in comparison with the control mice. Immunohistochemistry noted decreased Rnd3 expression along with increased α-smooth muscle actin (α-SMA) expression in diabetic mouse hearts (Figure 1D). These observations suggested a potential regulatory role of Rnd3 in DCM pathogenesis.

It is increasingly recognized that different types of cardiac cells possess distinct patterns of molecular expression and regulatory functions 24. To identify critical Rnd3-expressing cardiac cell type in DCM, cardiac myocytes (CD45^-^ PDGFR-α^-^ CD31^-^), fibroblasts (CD45^-^ PDGFR-α^+^), endothelial cells (CD45^-^ CD31^+^), and macrophages (CD45^+^ F4/80^+^) were separated using flow cytometry. Results of qPCR study demonstrated that expression level of Rnd3 was significantly higher in fibroblasts than other cell populations in control mice. Rnd3 expression was decreased in CFs, the levels of which were moderately decreased in cardiomyocytes and endothelial cells, and remained almost unchanged in macrophages of mice that underwent HFD-STZ treatment (Figure 1E). Rnd3 protein expression was significantly lower in CFs isolated from HFD-STZ-treated mice than those isolated from control mice. Meanwhile, α-SMA expression was higher in fibroblasts obtained from HFD-STZ-treated mice (Figure 1F-H). The aforementioned results strongly indicate that Rnd3 is associated with fibrosis and involved in DCM progression.

### Rnd3 downregulation in CFs exacerbates HFD-STZ-induced fibrosis and cardiac dysfunction

**Figure 2:**
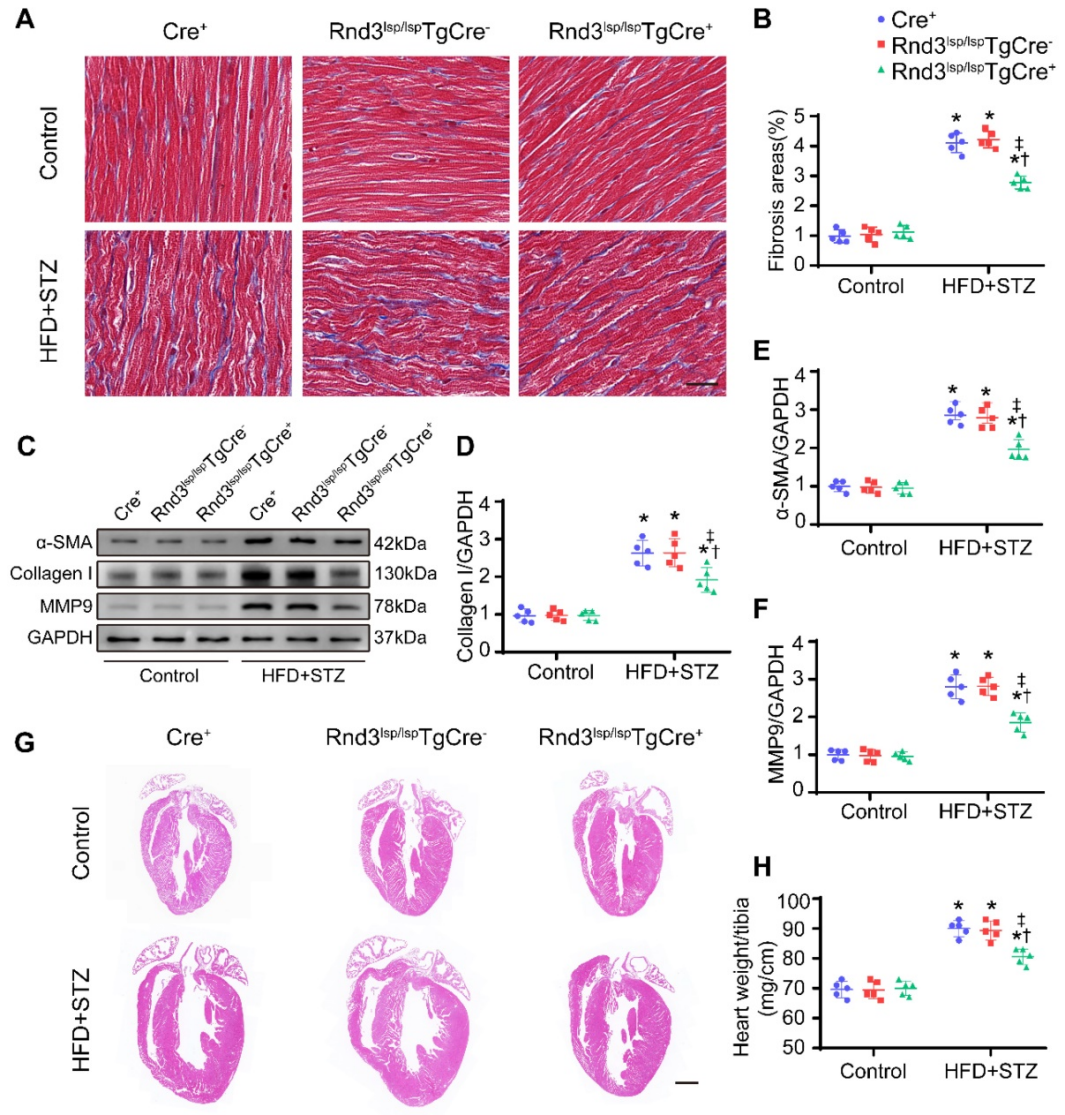
** Fibroblast-specific Rnd3 overexpression alleviates cardiac fibrosis and cardiac hypertrophy induced by HFD+STZ treatment. (A)** Representative Masson's trichrome staining of mouse hearts in different treatment groups; scale bars represent 20 µm. **(B)** Quantitative analysis of Masson's trichrome staining (n = 5). **(C-D)** Representative and pooled Western blot analysis of cardiac α-SMA expression in different treatment groups (n = 5). **(E)** qPCR analysis of cardiac collagen I expression in different treatment groups (n = 5). **(F)** Representative HE staining of mouse hearts in different treatment groups; scale bars represent 1 mm. **(G)** Quantitative analysis of heart weight in different groups (n = 5). *P < 0.05 *vs.* Control; ^†^P < 0.05 *vs.* Cre^+^ in HFD+STZ group; ^‡^P < 0.05 *vs.* Rnd3^fl/^flTgCre^-^ in HFD+STZ group.

**Figure 3:**
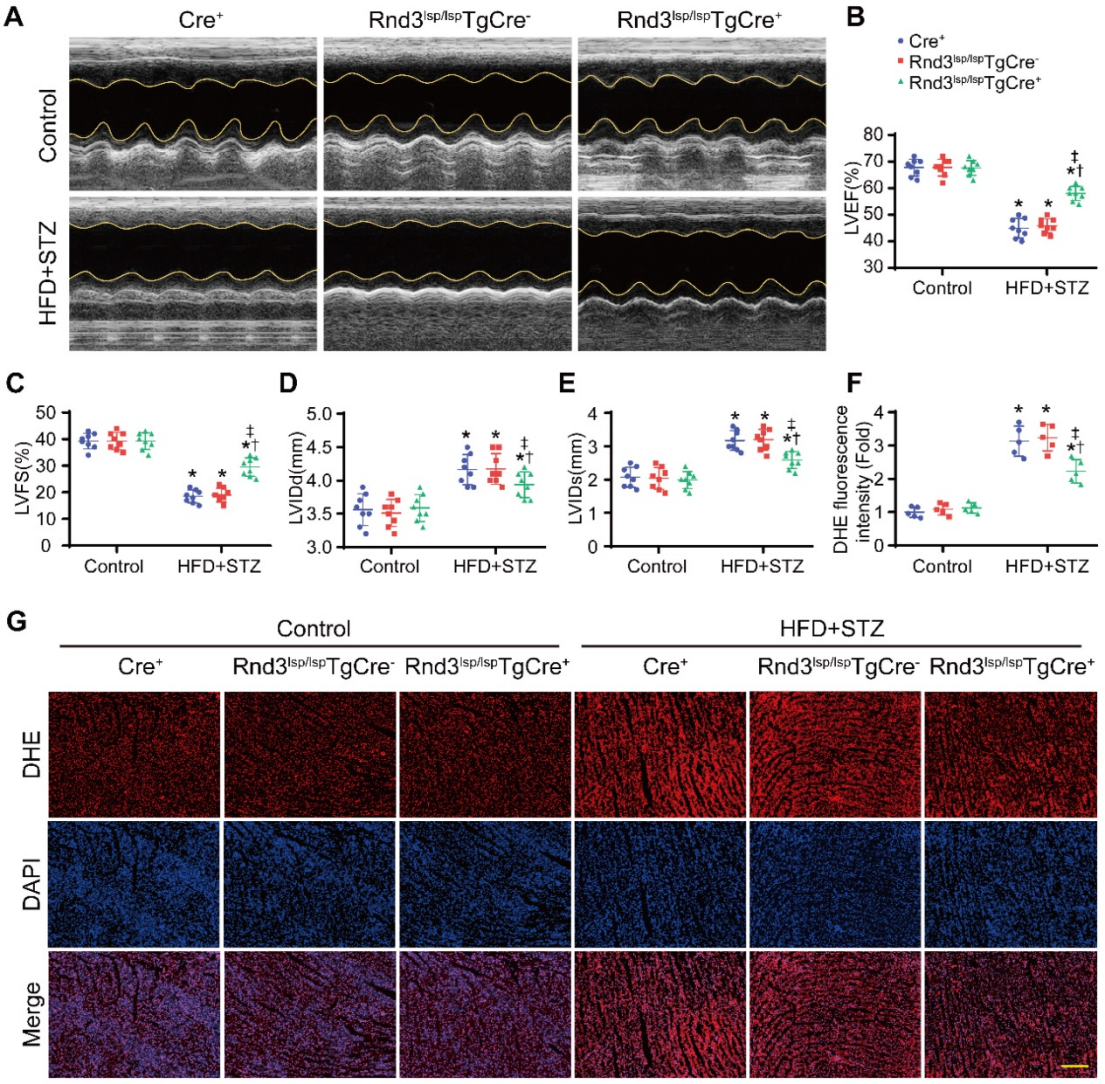
** Fibroblast-specific Rnd3 overexpression alleviates cardiac dysfunction and oxidative stress induced by HFD+STZ treatment. (A)** Cardiac function evaluated by M-mode echocardiograms (n = 8). **(B-E)** Quantitative analysis of cardiac function indices in different treatment groups (n = 8). **(F)** Quantitative analysis of reactive oxygen species (ROS) fluorescence intensity (n = 5). **(G)** ROS generation in mouse hearts from different treatment groups evaluated using DHE fluorescence; scale bars represent 50 µm. *P < 0.05 *vs.* Control; ^†^P < 0.05 *vs.* Cre^+^ in HFD+STZ group; ^‡^P < 0.05 *vs.* Rnd3^fl/fl^TgCre^-^ in HFD+STZ group.

We next assessed whether Rnd3 downregulation promoted or suppressed DCM. First, using a gain-of-function approach, a conditional fibroblast-specific Rnd3-transgenetic mouse model was generated. Fibroblast-specific Cre^+^ mice, Rnd3^lsp/lsp^TgCre^-^ mice, and Rnd3^lsp/lsp^TgCre^+^ mice were divided into HFD-STZ and control groups. Blood glucose level and body weight exhibited comparable increase in response to HFD-STZ challenge (Figure S1A). Rnd3 was significantly increased in cardiac fibroblasts (but not cardiomyocytes) from Rnd3^lsp/lsp^TgCre^+^mice compared with Cre^+^ and Rnd3^lsp/lsp^TgCre^-^ mice treated with HFD-STZ (Figure S1B-E). Masson's trichome staining revealed similar patterns of collagen deposition in Cre^+^, Rnd3^lsp/lsp^TgCre^-^, and Rnd3^lsp/lsp^TgCre^+^ mice in control group 24 weeks after HFD-STZ treatment. However, HFD-STZ-treated Rnd3^lsp/lsp^TgCre^+^ mice (Rnd3^lsp/lsp^TgCre^+^/HFD-STZ) showed a significant reduction of interstitial fibrosis (Figure 2A-B) and perivascular fibrosis (Figure S1F-G), compared with HFD-STZ-treated Cre^+^ mice (Cre^+^/HFD-STZ) and Rnd3^lsp/lsp^TgCre^-^ mice (Rnd3^lsp/lsp^TgCre^-^/HFD-STZ). Additionally, Rnd3^lsp/lsp^TgCre^+^/HFD-STZ mice displayed lower levels α-SMA, collagen I, and MMP9 protein expression (Figure 2C-F). Likewise, qPCR data also noted decreased mRNA level of α-SMA, collagen I, and MMP9 in Rnd3^lsp/lsp^TgCre^+^/HFD-STZ mice (Figure S1H-J). Morphological analysis revealed that Rnd3 overexpression attenuated HFD-STZ-induced ventricular dilatation and heart weight-tibial length ratio, compared with Cre^+^ and Rnd3^lsp/lsp^TgCre^-^ mice (Figure 2G-H). Rnd3^lsp/lsp^TgCre^+^/HFD-STZ mice also exhibited a significant improvement in cardiac function, exemplified by increased left ventricular ejection fraction (LVEF, Rnd3^lsp/lsp^TgCre^+^/HFD-STZ *vs.* Rnd3^lsp/lsp^TgCre^-^/HFD-STZ, 58.2% *vs.* 46.8%; Rnd3^lsp/lsp^TgCre^+^/HFD-STZ *vs.* Rnd3^lsp/lsp^TgCre^+^/Control, 58.2% *vs.* 68.6%), and left ventricular fractional shortening (LVFS), a decrease in left ventricular diastolic internal dimension (LVIDd) and left ventricular systolic internal dimension (LVIDs) (Figure 3A-E). Rnd3 overexpression reduced the reactive oxygen species (ROS) generation, a cardinal mediator of fibrosis in diabetes mellitus 25, in fibroblasts, (Figure 3F-G). These findings suggested that Rnd3 alleviates DCM development likely through regulation of ROS generation.

**Figure 4:**
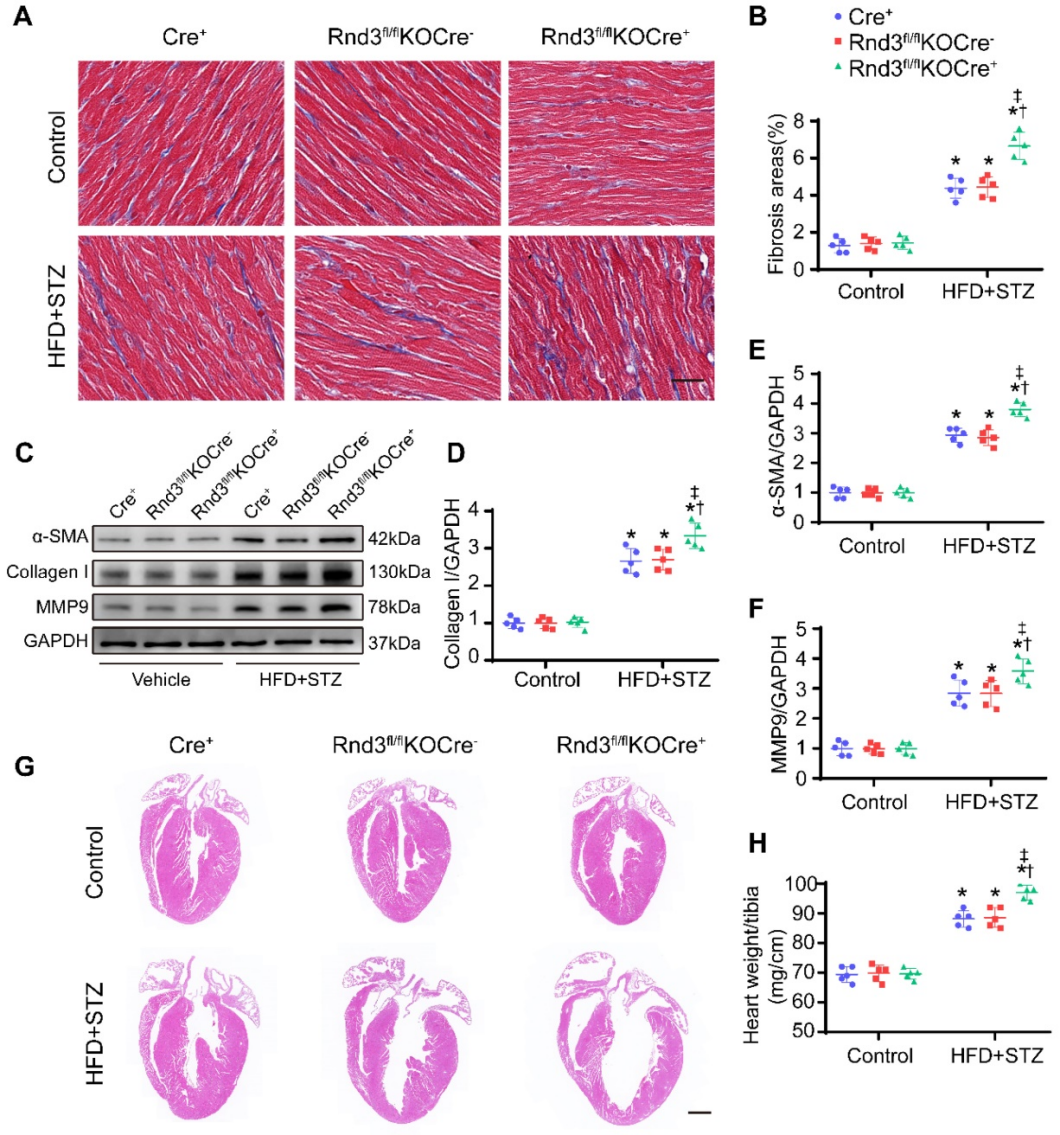
** Fibroblast-specific Rnd3 knockout exacerbates cardiac fibrosis and cardiac hypertrophy induced by HFD+STZ treatment. (A)** Collagen deposition in mouse hearts evaluated using masson's trichrome staining in different treatment groups; scale bars represent 20 µm (n = 5). **(B)** Quantitative analysis of Masson's trichrome staining (n = 5). **(C-D)** Representative and pooled Western blot analysis of cardiac α-SMA expression in different groups (n = 5). **(E)** qPCR analysis of cardiac collagen I expression in different treatment groups (n = 5). **(F)** Representative HE staining of mouse cardiac tissues in different treatment groups; scale bars represent 1 mm. **(G)** Quantitative analysis of heart weights of mice in different groups (n = 5). *P < 0.05 *vs.* Control; ^†^P < 0.05 vs. Cre^+^ in HFD+STZ group; ^‡^P < 0.05 vs. Rnd3^fl/fl^KOCre^-^ in HFD+STZ group.

**Figure 5:**
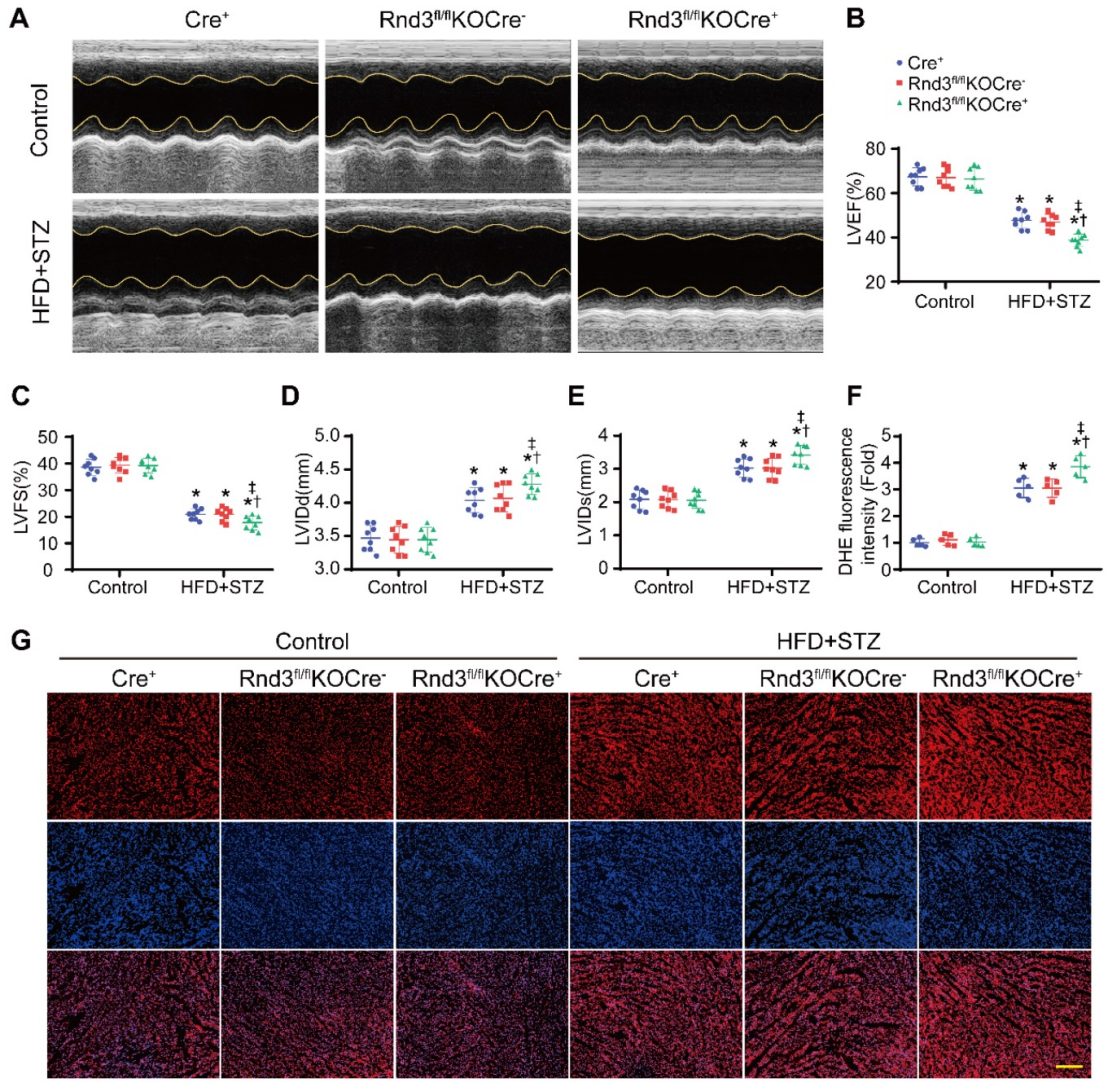
** Fibroblast-specific Rnd3 knockout exacerbates cardiac dysfunction and oxidative stress induced by HFD+STZ treatment. (A)** Cardiac function evaluated by M-mode echocardiograms. **(B-E)** Quantitative analysis of cardiac function indices in different groups (n = 8). **(F)** Quantitative analysis of reactive oxygen species (ROS) generation fluorescence intensity (n = 5). **(G)** ROS in hearts from different mouse groups evaluated using DHE fluorescence; scale bars represent 50 µm. *P < 0.05 *vs.* Control; ^†^P < 0.05 *vs.* Cre^+^ in HFD+STZ group; ^‡^P < 0.05 *vs.* Rnd3^fl/fl^KOCre- in HFD+STZ group.

Next, fibroblast-specific Rnd3 knockout mice were generated. Fibroblast-specific Cre^+^ mice, Rnd3^fl/fl^KOCre^-^ mice, and Rnd3^fl/fl^ KOCre^+^ mice were subjected to HFD-STZ for 24 weeks. HFD-STZ treatment induced a similar rise in body weight and blood glucose levels in Cre^+^, Rnd3^fl/fl^KOCre^-^, and Rnd3^fl/fl^KOCre^+^ mice (Figure S2A). Knockout of Rnd3 in CFs and cardiomyocytes were detected by western blot (Figure S2B-E). Cardiac tissues from HFD-STZ mice displayed pronounced collagen deposition compared with the control group. However, collagen deposition was significantly higher in Rnd3^fl/fl^KOCre^+^/HFD-STZ mice than that in Cre^+^/HFD-STZ and Rnd3^fl/fl^KOCre^-^/HFD-STZ mice (Figure 4A-B, Figure S2F-G). Consistent with cardiac fibrosis phenotypes in Rnd3^fl/fl^KOCre^+^/HFD-STZ mice, protein and mRNA level of α-SMA, collagen I, and MMP9 were upregulated in fibroblast-specific Rnd3 knockout mouse hearts (Figure 4C-F, Figure S2H-J). Furthermore, Rnd3^fl/fl^KOCre^+^/HFD-STZ mice exhibited greater ventricular dilatation and a higher heart weight-tibial length ratio (Figure 4G-H). Meanwhile, fibroblast-specific Rnd3 knockout worsened cardiac function determined using echocardiography. In comparison with Cre^+^/HFD-STZ and Rnd3^fl/fl^KOCre^-^ mice, Rnd3^fl/fl^KOCre^+^ mice exhibited lowered levels of LVEF and LVFS, accompanied by a higher LVIDd and LVIDs (Figure 5A-E). Mechanistically, Rnd3 deficiency contributed to DCM progression likely through elevated ROS generation (Figure 5F-G).

Taken together, these results demonstrated a protective role for Rnd3 against DCM, while Rnd3 downregulation exacerbates ventricular remodeling, cardiac fibrosis, and dysfunction in diabetic hearts.

### Rnd3 regulates *in vitro* CF activation

**Figure 6:**
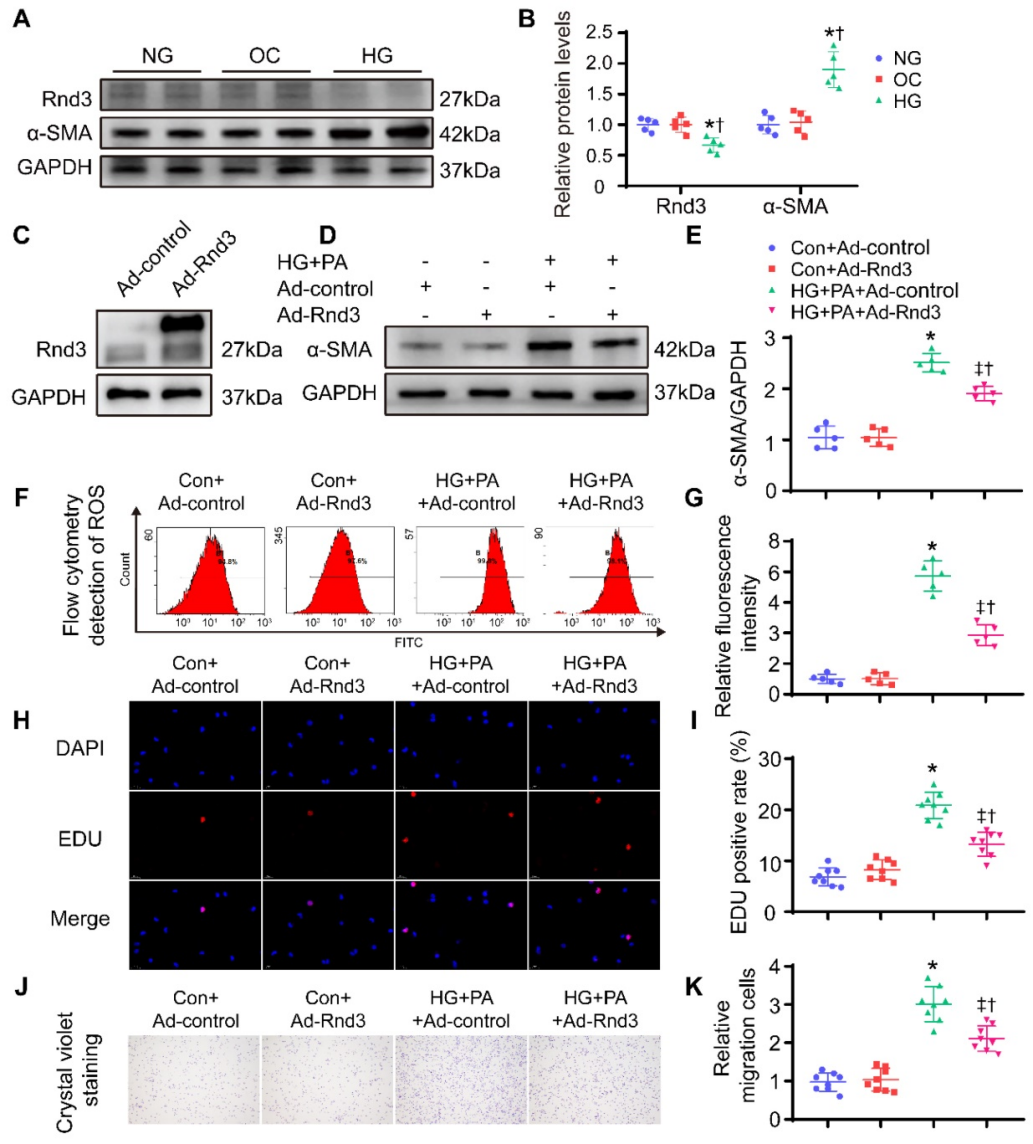
** Rnd3 overexpression reduces proliferation, migration, and reactive oxygen species (ROS) generation in cardiac fibroblasts (CFs) subjected to HG+PA treatment. (A-B)** Rnd3 and α-SMA expression in CFs evaluated by Western blot analysis. *P < 0.05 vs. NG; ^†^P < 0.05 vs. OC. **(C)** Transfection efficiency of adenoviruses evaluated using western blot. **(D-E)** Western blot and α-SMA in CFs with the indicated treatment (n = 5). **(F-G)** Flow cytometry and associated quantitative analysis of ROS generation in different treatment groups (n = 5). **(H)** The effect of Rnd3 overexpression on CF proliferation evaluated using EdU assay (n = 8). **(I)** Quantitative analysis of the proliferation assay in different treatment groups (n = 8). **(J-K)** Representative crystal violet staining and quantitative analysis in transwell chemotactic assay of CFs treated as indicated (n = 8). *P < 0.05 *vs.* Con+Ad-control;^ †^P < 0.05 *vs.* Con+Ad-Rnd3; ^‡^P < 0.05 *vs.* HG+PA+Ad-control.

To further discern the role of Rnd3 in fibrosis, CFs were isolated from neonatal mice and cultured at different concentrations of glucose (5; 25; 30; 35 mmol/L) and palmitic acid (PA; 500 μmol/L) to induce glycotoxicity and lipotoxicity *in vitro* (Figure S3A-B). High glucose (HG; 35 mmol/L) and PA treatment significantly enhanced α-SMA expression and decreased Rnd3 expression, consistent with the *in vivo* results. Furthermore, the mannitol at the same concentration (5.5 mmol/L glucose and 29.5mmol/L mannitol) has no effect on fibroblast activation and Rnd3 expression level (Figure 6A-B). Then, CFs were infected with an Rnd3-overexpressing adenovirus (Ad-Rnd3), and western blot analysis was used to evaluate transfection efficiency of the adenovirus (Figure 6C). Rnd3 overexpression effectively prevented activation of CFs underwent HG-PA treatment (Figure 6D-E, Figure S3E). Specifically, Ad-Rnd3 decreased ROS generation in CFs (Figure 6F-G). EdU proliferation assay revealed a 7.6% positive rate in control group which was not significantly affected by Ad-Rnd3 transfection. Conversely, the HG-PA group displayed a positive rate of 21.3% that was decreased to 13.6% by Ad-Rnd3 transfection (Figure 6H-I). Similarly, CCK8 assay showed that overexpression of Rnd3 significantly decreased proliferation ability of CFs under HG-PA (Figure S3F). Rnd3 overexpression also reduced fibroblast migration in transwell chamber in cells treated with HG-PA (Figure 6J-K).

**Figure 7:**
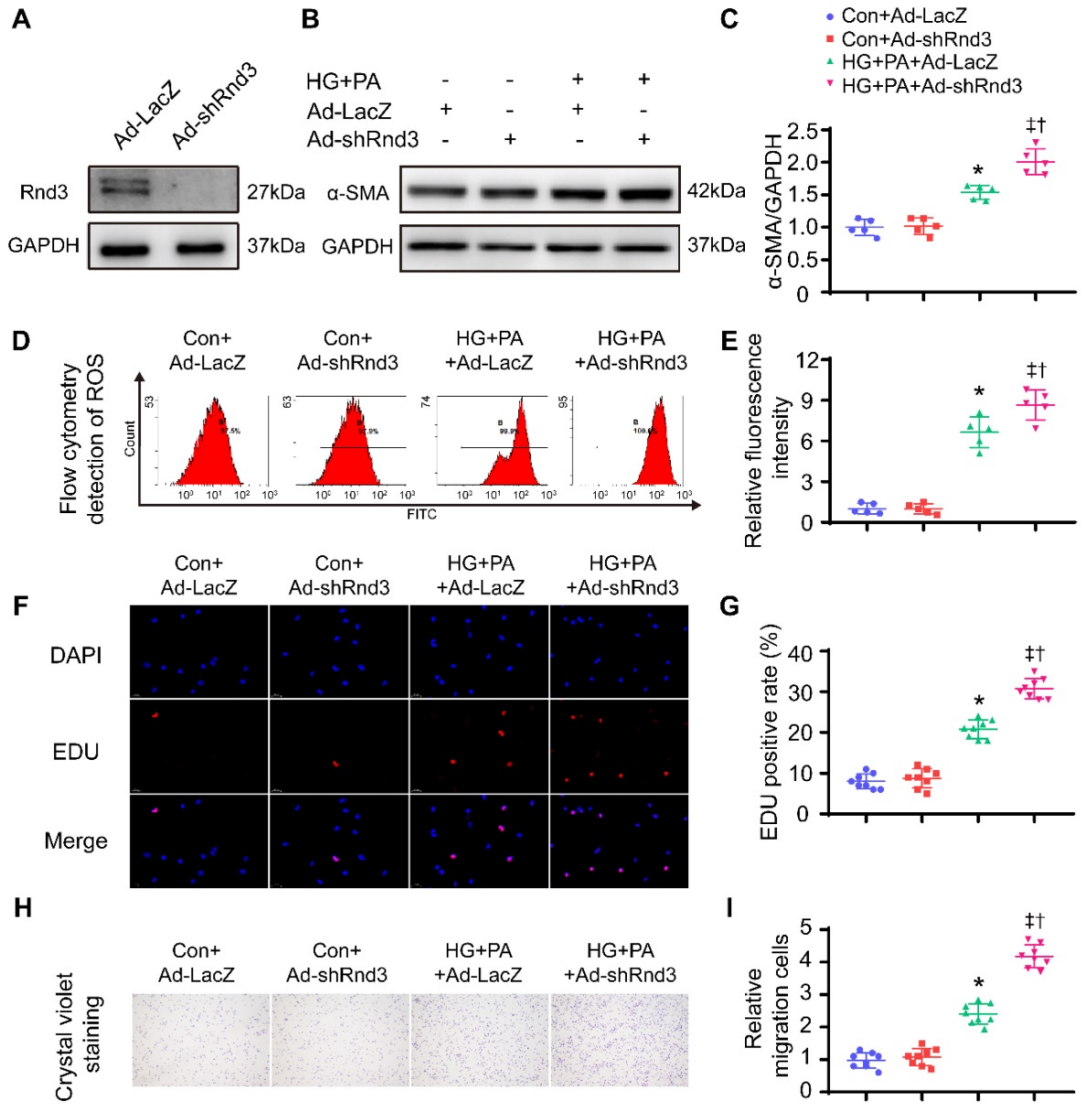
** Rnd3 knockout contributes to proliferation, migration, and reactive oxygen species (ROS) generation in cardiac fibroblasts (CFs) subjected to HG+PA treatment. (A)** Transfection efficiency of adenovirus evaluated by western blot analysis. **(B-C)** Representative and quantitative Western blot analysis of α-SMA in CFs from various treatment groups (n = 5). **(D-E)** Flow cytometry and associated quantitative analysis of ROS generation in CFs with various treatment (n = 5). **(F)** The influence of Rnd3 knockdown on CF proliferation evaluated using EdU assay (n = 8). **(G)** Quantitative analysis of the proliferation assay in different groups (n = 8). **(H-I)** Representative crystal violet staining and quantitative analysis in transwell chemotactic assay of CFs with various treatments (n = 8). *P < 0.05 vs. Con+Ad-LacZ; ^†^P < 0.05 *vs.* Con+Ad-shRnd3; ‡P < 0.05 vs. HG+PA+Ad-LacZ.

Conversely, Rnd3-knockdown adenoviruses were also used to infect CFs, Western blot analysis was used to evaluate transfection efficiency of adenovirus (Figure 7A). As shown in Figure 7B-C and Figure S4A, Rnd3 knockdown significantly enhanced the protein and mRNA level of α-SMA in CFs under HG-PA. Flow cytometry data showed that HG-PA evoked a remarkable increase in ROS production compared with control group, whereas Rnd3 knockdown exacerbated ROS production in fibroblasts (Figure 7D-E). Proliferation (Figure 7F-G, Figure S4B) and migration (Figure 7H-I) potential of CFs were also accentuated by Rnd3 knockdown in the face of HG-PA challenge, without notable change in control group. Taken together, Rnd3 knockdown exacerbated HG-PA-evoked CF activation in association with ROS production.

### Rnd3 inhibits TGF-β and Notch signaling to regulate fibrotic response

**Figure 8:**
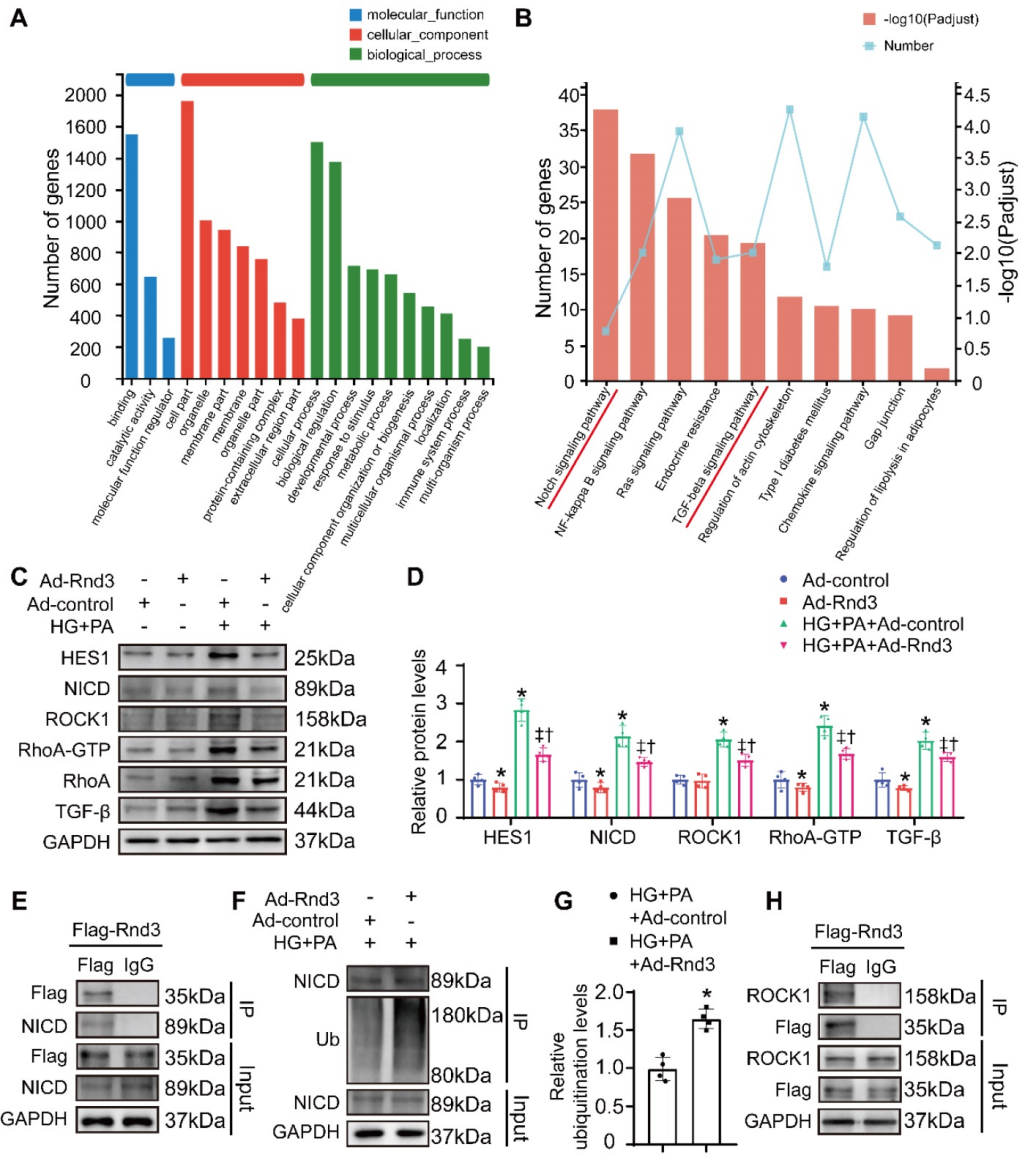
** Rnd3 inhibits TGF-β and Notch signaling to regulate fibrotic response under HG+PA. (A)** Gene ontology analysis of RNA-sequencing data from control and Ad-Rnd3 cardiac fibroblasts (CFs). **(B)** Kyoto Encyclopedia of Genes and Genomes (KEGG) pathway analysis showing major signaling pathways in control and Ad-Rnd3 CFs. **(C)** Western blot analysis of Hes1, NICD, ROCK1, RhoA-GTP, RhoA, and TGF-β1 expression in CFs treated as indicated. **(D)** Quantitative analysis of the protein levels of Hes1, NICD, ROCK1, RhoA-GTP, RhoA, and TGF-β1 in CFs treated as indicated (n = 4). *P < 0.05 vs. Ad-control; ^†^P < 0.05 *vs.* Ad-Rnd3; ^‡^P < 0.05 *vs.* HG+PA+Ad-control. **(E)** Interaction between Rnd3 and NICD demonstrated using co-immunoprecipitation. **(F)** Co-immunoprecipitation and Western blot analysis of ubiquitination level of NICD in different treatment groups. **(G)** Quantitative analysis of the ubiquitination level of NICD in different groups (n = 3). *P < 0.05 vs. HG+PA+Ad-control. **(H)** Interaction between Rnd3 and ROCK1 demonstrated using Co-immunoprecipitation.

To explore possible mechanisms through which Rnd3 regulates fibroblast activation, RNA-seq analysis was performed in fibroblasts infected with or without Rnd3-overexpressing adenovirus in the face of HG-PA challenge. A total of 968 differentially expressed genes (with a |log2FC|≥1 and p value<0.05) were identified (Table S1). GO analysis showed that Rnd3-related genes were primarily categorized into cellular process, biological regulation, binding, and cell components (Figure 8A). Comparative KEGG pathway analysis revealed that the major affected pathways encompassed Notch signaling pathway, NF-κB signaling pathway, Ras signaling pathway, chemokine signaling pathway, and TGF-β signaling pathway, among others (Figure 8B). Among different signaling pathways, we focused on the Notch and TGF-β signaling pathways.

**Figure 9:**
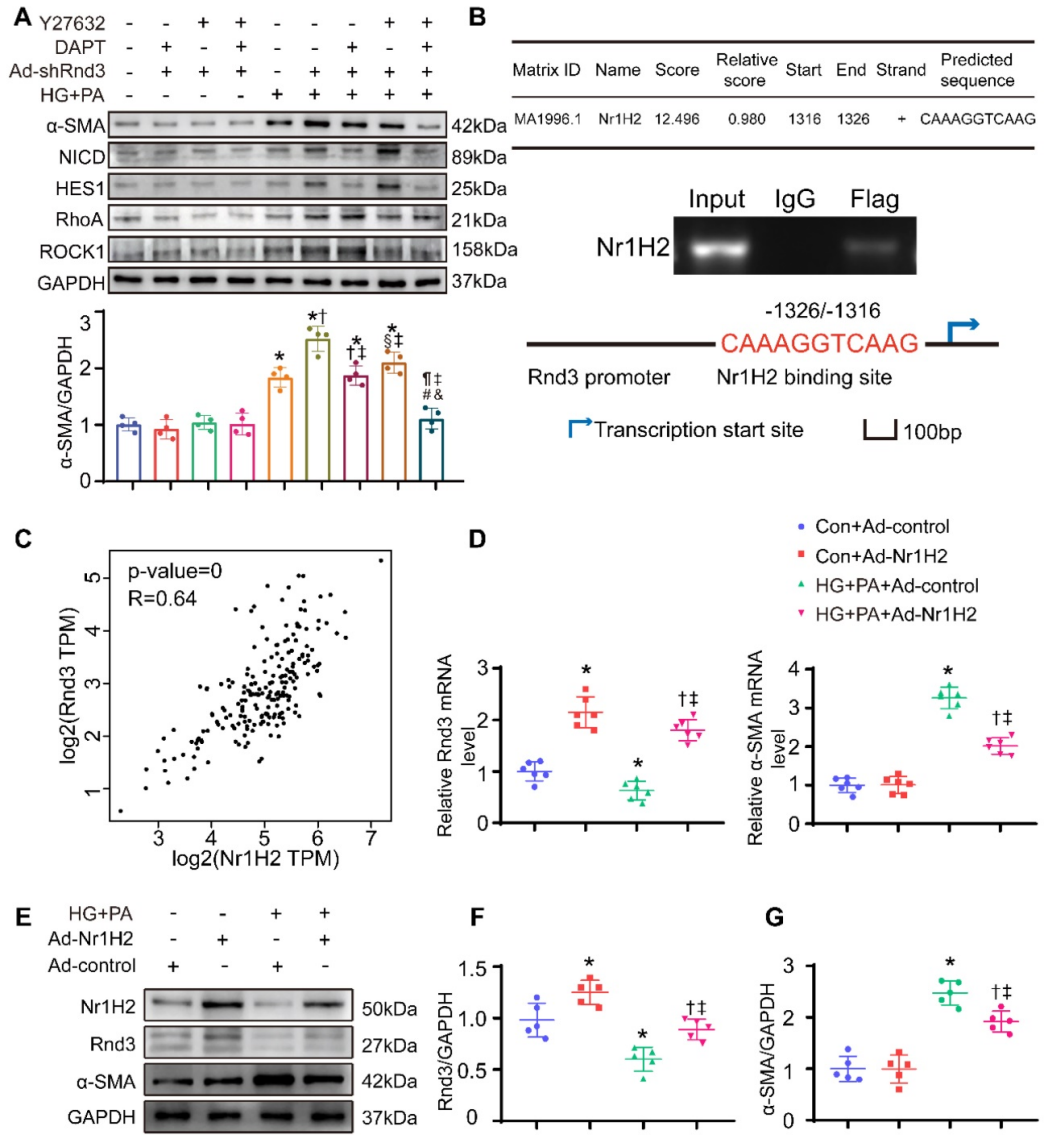
** Nr1H2 directly upregulates Rnd3 transcription in cardiac fibroblasts (CFs). (A)** Western blot analysis of α-SMA, Hes1, NICD, ROCK1, and RhoA expression in CFs treated as indicated (n = 4). *P < 0.05 *vs.* Control; ^†^P < 0.05 *vs.* DAPT+Ad-shRnd3; ^§^P < 0.05 *vs.* Y27632+Ad-shRnd3; ^¶^P < 0.05 *vs.* HG+PA; ^‡^P < 0.05 *vs.* HG+PA+Ad-shRnd3; ^#^P < 0.05 *vs.* DAPT+HG+PA+Ad-shRnd3; ^&^P < 0.05 *vs.* Y27632+HG+PA+Ad-shRnd3. **(B)** Chromatin immunoprecipitation analysis of Nr1H2 binding to the Rnd3 promoter in CFs with indicated treatments. **(C)** Pearson's correlation analysis of Rnd3 and Nr1H2 expression in cardiac tissues using the GEPIA program. **(D)** qPCR analysis of Rnd3 and α-SMA expression in heart tissues after indicated treatments (n = 6). **(E-G)** Western blot and quantitative analysis of Rnd3 and α-SMA expression in CFs with the indicated treatments (n = 5). *P < 0.05 *vs.* Con+Ad-control; ^†^P < 0.05 vs. Con+Ad-Nr1H2; ^‡^P < 0.05 vs. HG+PA+Ad-control.

Rnd3 is a critical regulator of Notch signaling 26, 27, and the interaction between Rho GTPase and TGF-β signaling was widely reported 28, 29. Here, we assessed the protein levels of genes related to these signaling pathways. As expected, protein expression of the Notch intracellular domain (NICD) and the Notch signaling target gene Hes1, as well as TGF-β signaling related genes TGF-β1, RhoA, and Rock1, were markedly decreased in CFs treated with Ad-Rnd3 in the conditioned medium (Figure 8C-D). Additionally, immunoprecipitation assays revealed the interaction between Rnd3 and NICD in CFs treated with Ad-Rnd3-Flag in conditioned medium, and Rnd3 overexpression substantially promoted protein ubiquitination of NICD (Figure 8E-G). Specifically, the interaction between Rnd3 and Rock1 was also observed in CFs (Figure 8H). To verify the regulatory role of Rnd3 in signaling pathways, CFs infected with the vector or Rnd3-knockdown adenovirus (Ad-shRnd3) were treated with antagonists against Notch1 (DAPT, MCE, 200 nM) and Rock1 (Y27632, MCE, 200 μM). Inhibition of Notch1 and Rock1 reversed upregulation ofα-SMA induced by Ad-shRnd3. Inhibition of both Notch1 and Rock1 further decreased level of α-SMA compared with that from inhibition of Notch1 or Rock1 alone, indicating combined action of Notch and TGF-β signaling in cardiac fibrogenesis in response to Rnd3 knockdown (Figure 9A).

To verify the mechanisms *in vivo*, CFs were isolated from Cre^+^ mice, Rnd3^lsp/lsp^TgCre^-^ mice, and Rnd3^lsp/lsp^TgCre^+^ mice in control and HFD-STZ groups as well as cultured cells under normal glucose for 18 hours. Then, activation status and related pathways were detected using Western blot analysis. As shown in Figure S3C-D, HFD-STZ induced a significant rise of α-SMA expression in CFs, whereas CFs from Rnd3^lsp/lsp^TgCre^+^ mice displayed a significant drop in α-SMA expression compared with Cre^+^ and Rnd3^lsp/lsp^TgCre^-^ mice in HFD-STZ group. Notch and TGF-beta/ROCK signaling in CFs were also overtly activated by HFD-STZ, while overexpression of Rnd3 substantially inhibited activation. Conversely, knockout of Rnd3 exacerbated activation of CFs and related signaling (Figure S4C-D). These findings indicated that Rnd3 inhibited both Notch and TGF-β signaling to regulate fibrotic response.

### Nr1H2 directly upregulates Rnd3 transcription in CFs

Last but not least, the mechanism underlying reduction of Rnd3 expression was explored in CFs subjected to HG-PA challenge. Putative transcription factors were predicted using the Jaspar program (http://jaspar.genereg.net/), and the top 10 putative transcription factors of Rnd3 (Table S2) were determined using ChIP. Nr1H2 directly binds to the promoter of Rnd3 (-1326/-1316) (Figure 9B). Moreover, the putative correlation between Rnd3 and Nr1H2 expression was examined using the GEPIA software (http://gepia.cancer-pku.cn/). As indicated in Figure 9C, Rnd3 and Nr1H2 expression showed a positive correlation (p-value=0, R=0.64) in cardiac tissues. As a piece of additional evidence, Nr1H2-overexpressing adenovirus (Ad-Nr1H2) significantly enhanced Rnd3 expression at both mRNA and protein levels, and Ad-Nr1H2 also reversed changes in α-SMA expression in fibroblasts that underwent the indicated treatments (Figure 9D-G).

**Figure 10:**
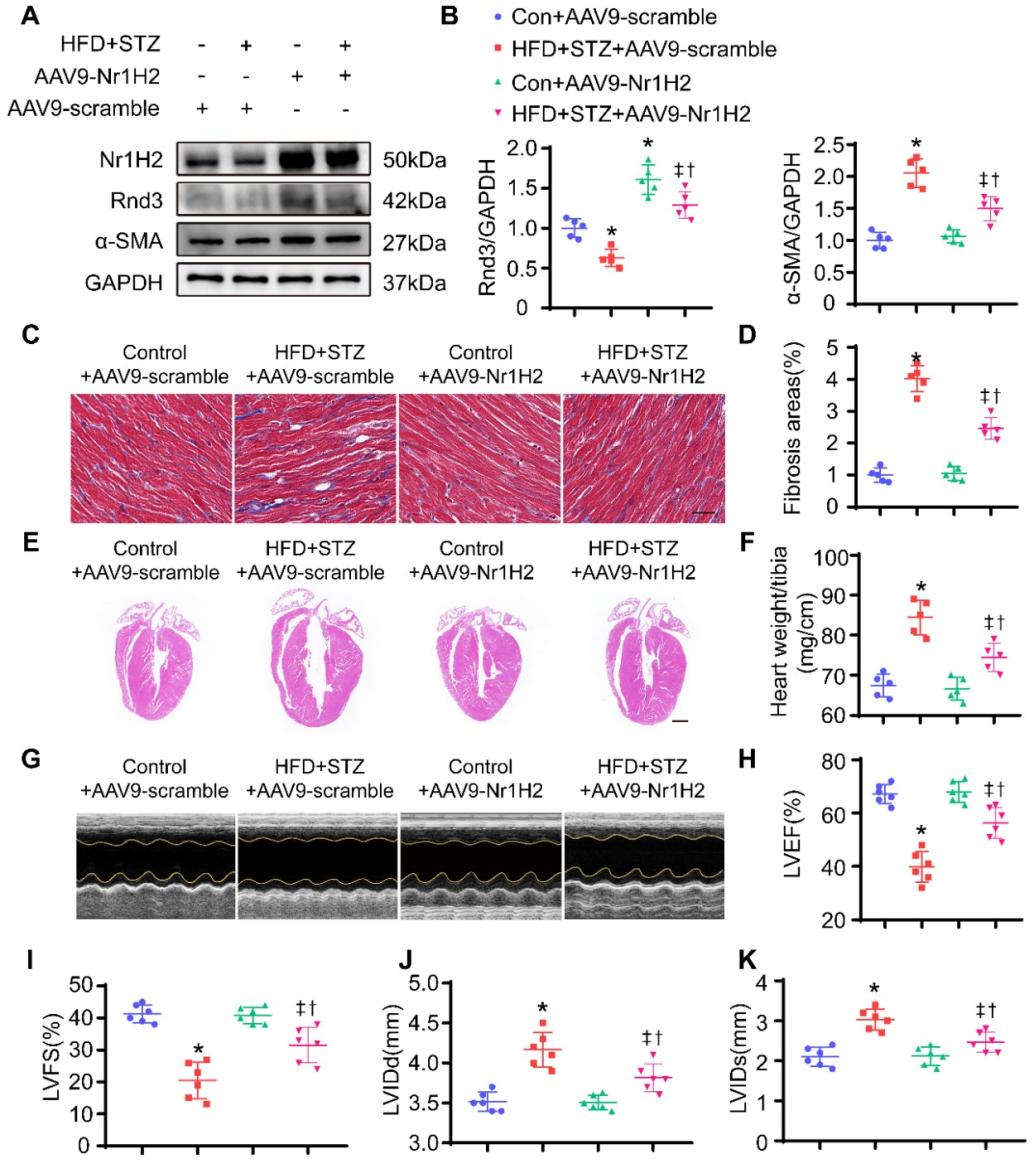
** Nr1H2 overexpression alleviates cardiac function, fibrosis, and cardiac hypertrophy induced by HFD+STZ treatment. (A)** Western blot analysis of α-SMA, Nr1H2, and Rnd3 in various treatment groups (n = 5). **(B)** Quantitative analysis of the protein levels of α-SMA, Nr1H2, and Rnd3 in various treatment groups (n = 5). **(C)** Representative images of Masson's trichrome staining of heart tissues from various treatment groups; scale bars represent 20 µm. **(D)** Quantitative analysis of interstitial Masson's trichrome staining (n = 5). **(E)** Representative HE staining of heart tissues in various treatment groups; scale bars represent 1 mm. **(F)** Quantitative analysis of the ratio of heart weight to tibia length in different groups (n = 5). **(G)** Cardiac function evaluated using M-mode echocardiograms. **(H-K)** Quantitative analysis of cardiac function indices in various treatment groups (n = 6). *P < 0.05 vs. Con+AAV9-scramble; ^†^P < 0.05 *vs.* HFD+STZ+AAV9-scramble; ^‡^P < 0.05 *vs.* Con+AAV9-Nr1H2.

*In vivo*, HFD-STZ-treated mice were injected with AAV9. AAV9-Nr1H2 or AAV9-scramble was injected 1 week following final STZ injection. Four weeks later, expression of Nr1H2 and development of cardiac phenotype were assessed. As shown in Figure 10A, Nr1H2 overexpression was confirmed using Western blot. Rnd3 expression was overtly elevated in AAV9-Nr1H2-infected HFD-STZ-treated mouse hearts compared with AAV9-scramble-infection in HFD-STZ mice. Meanwhile, HFD-STZ reduced the level of Nr1H2, while Nr1H2 overexpression reduced α-SMA expression in HFD-STZ mouse hearts (Figure 10B, Figure S4E-F). Masson trichome staining also showed that AAV9-Nr1H2 injection suppressed interstitial and perivascular fibrosis in hearts from HFD-STZ-treated mice compared with AAV9-scramble injection (Figure 10C-D, Figure S4G-H). Heart weight and ventricular dilatation were significantly alleviated by Nr1H2 overexpression in HFD-STZ-treated mice (Figure 10E-F). More importantly, AAV9-Nr1H2 injection improved cardiac function compared with AAV9-scramble injection, exemplified by an increase in LVEF and LVFS, a decrease in LVIDd and LVIDs (Figure 10G-K). Collectively, our data indicate that Rnd3 transcription in CFs is controlled by Nr1H2.

## Discussion

The salient findings from our present study depicted a major role for Rnd3 in cardiac remodeling in diabetic hearts. Our results noted downregulated Rnd3 in hearts from HFD-STZ-treated mice, while CFs were identified as the primary Rnd3-expressing cell type under Nr1H2 regulation. Next, Rnd3-expressing CFs imparted a substantial protective effect against diabetic ventricular remodeling. In contrast, Rnd3 expression deficiency accentuated fibrosis and cardiac dysfunction in diabetes. Third, Rnd3 ameliorated diabetic ventricular remodeling via suppressing Notch and TGF-β signaling. These findings favor that fibroblast-specific activation of Rnd3 preserves against cardiac remodeling in DCM, indicating promises of targeting Rnd3 in the management of DCM.

Our findings demonstrated that Rnd3 was downregulated in the hearts of HFD-STZ-treated mice, suggesting possible clinical implication of Rnd3 downregulation in DCM. Cardiac Rnd3 mRNA expression was reported significantly lower in human heart failure, according to a microarray screening study (Profile GDS651/212724_at/RND3 in NCBI GEO profiles). Along the same line, Rnd3^+/-^ haploinsufficiency predisposes animal hearts to pressure overload, although it remains elusive with regards to the etiological implication of Rnd3 downregulation and major cell type 10. To obtain robust evidence on the origin of Rnd3 to DCM, we identified CFs as the major cell type expressing Rnd3 in cardiac tissues. In this regard, the predominance of Rnd3-expressing CFs could be attributed to the biological function of Rnd3 in cytoskeletal regulation and the physiological properties of fibroblasts 30. We adopted two approaches to determine the role of Rnd3 in CFs. Results from fibroblast-specific Rnd3-overexpressing mice showed that CF-specific Rnd3 overexpression attenuated HFD-STZ-induced cardiac remodeling and contractile defect, while fibroblast-specific Rnd3 knockout accentuated HFD-STZ-induced cardiac remodeling. Evidence from these loss- and gain-of-function studies supported that Rnd3 downregulation sensitizes the heart to diabetic challenge, consolidating the protective role of Rnd3 against diabetic cardiac remodeling.

Under detrimental biochemical conditions, fibrotic growth factors, cytokines, and neurohumoral pathways trigger fibrotic intracellular signaling cascades that eventually turn on CFs. In this study, both *in vitro* and *in vivo* models were employed to simulate the abovementioned phenomenon. First, we simulated activation of CFs *in vivo* under HG-PA treatment prior to transcriptomics and ChIP analyses in Rnd3-overexpressing fibroblasts. Results of the transcriptomics study revealed that Notch signaling and TGF-β signaling may serve as targets of Rnd3 for suppressing fibroblast activation. Increasing evidence indicated that Notch signaling is of vital importance in fibrosis in chronic fibroproliferative diseases affecting various organs and tissues 31, 32. Lin and colleagues reported that Rnd3 deficiency suppressed NICD degradation, eventually upregulating Notch activity and promoting aberrant growth of aqueduct ependymal cells 26. Our results show that Rnd3 physically interacted with NICD to promote NICD ubiquitination. Therefore, forced induction of Rnd3 expression would repress Notch signaling through post-translational modification, leading to inhibition of CF activation.

ROCK1, the key downstream target of RhoA, participates in numerous physiological functions related to cytoskeletal rearrangement 33, 34. ROCK1 is also known to enhance NADPH oxidase expression, contributing to ROS production. Rnd3 has been shown to attenuate oxidative stress in vascular smooth muscle cells through inhibition of ROCK1 signaling 13. Rnd3 was also noted to regulate ROS production in preeclampsia, supported by the observation that Rnd3 overexpression partially rescued oxidative stress in primary trophoblasts in the onset of preeclampsia 35. Our findings supported a regulatory role of Rnd3 in ROS generation. As a key representative fibrotic growth factor, TGF-β participates in fibrosis through RhoA/ROCK1 28, 36. In addition, RhoA/ROCK1 can also participate in feedback activation to TGF-β signals 29. The antagonistic effect of Rnd3 on RhoA/ROCK1 effectively interrupts the feedback loop. Therefore, loss of Rnd3 expression contributes to ROS generation and fibroblast activation.

Notably, Nr1H2 binds directly to the Rnd3 promoter. Nr1H2, a ubiquitously expressed transcription factor belonging to the nuclear hormone receptor superfamily, regulates glucolipid metabolism and inflammatory responses in a variety of diseases 37-39. Human Nr1H2 gene is controlled by glucose, and serves as an attractive drug target in diabetic injury 40, 41. Our results demonstrated that Nr1H2 exerts a cardioprotective effect against DCM through Rnd3 (at least in part) *in vivo* and vitro.

Diabetes is well perceived to promote injury of cardiomyocytes and endothelial cells. In response to diabetic insult, cardiac fibroblasts get activated to play a vital role in wound healing involving production and deposition of matrix proteins such as collagen. Our data demonstrated improvement in interstitial and perivascular fibrosis, myocardial structure and function upon Rnd3 over-expression. It is noteworthy that CFs are not the only cell type afflicted in DCM. For example, CMs and ECs are also negatively affected by diabetes and hyperglycemia. The possibility of Rnd3-dependent paracrine effects of CFs on CMs and ECs cannot be excluded at this time. This is in line with the observation that Rnd3 is considered a novel factor in the regulation of intracellular calcium homeostasis in CMs 11 and inflammation 12. In particular, Rnd3 promotes recovery of endothelial barrier during inflammatory challenge and participates in angiogenesis during heart failure 42, 43. Rnd3-dependent cardiac fibroblast response is only one (important) aspect of the complex pathophysiology of DCM although contribution from CMs and ECs warrants future work. Considering that restored level of Rnd3 serves as a protective mechanism in heart diseases, development of medications to jack up Rnd3 signaling may be a promising therapeutic approach for DCM and associated cardiac diseases.

It should be mentioned that our study suffers from several limitations. While db/db, ob/ob, HFD/STZ, and HFD models 14, 44 are commonly used as T2DM mouse models, the critical role of Rnd3 in DCM should be validated in human diabetes and other murine models of diabetes. Although our results suggest that Nr1H2 restored Rnd3 level and alleviated cardiac remodeling and fibrosis, the AAV-Nr1H2 used functions as a broad spectrum promoter, overexpression of Nr1H2 and Rnd3 in non-CFs including endothelial cells and cardiomyocytes may also contribute to the protective effects. In addition, other pathways enriched using KEGG analysis, such as the NF-κB signaling pathway, may also exhibit profound interaction with ROS generation and should not be ignored. In conclusion, findings of this study indicate a novel mechanism for alleviating diabetic cardiomyopathy and highlight the therapeutic promises of targeting Rnd3 in the management of DCM.

## Supplementary Material

Supplementary figures and tables.

Supplementary table.

## Abbreviations

DCM: diabetic cardiomyopathy
ECM: extracellular matrix
CF: cardiac fibroblast
Rnd3: Rho family GTPase 3
T2DM: type 2 diabetes mellitus
STZ: streptozotocin
HG: high levels of glucose
PA: palmitic acid
EdU: 5-ethynyl-2'-deoxyuridine
DHE: Dihydroethidium
ROS: reactive oxygen species
RhoA: Ras homolog gene family member A
ROCK1: Rho-associated kinase 1
NICD: Notch intracellular domain
HES1: hes family bHLH transcription factor 1
Nr1H2: Nuclear Receptor Subfamily 1 Group H Member 2
GO: gene ontology
KEGG: Kyoto Encyclopedia of Genes and Genomes
