# Supplementary material for: Fibroblast-specific activation of Rnd3 protects against cardiac remodeling in diabetic cardiomyopathy via suppression of Notch and TGF-β signaling
Source: Theranostics. 2022 Oct 17;12(17):7250–66. doi: 10.7150/thno.77043 (PMC9691359; doi:10.7150/thno.77043)
Supplement: Supplementary file 1 — Supplementary figures and tables. [file thnov12p7250s1.pdf]

# Supplemental Data

## Detailed Methods

**The 5-Ethynyl-2′-Deoxyuridine (EdU) Cell Proliferation Assay**

Cell proliferation was detected using an EDU kit (Beyotime Biotechnology, Shanghai, China) according to the manufacturer’s instructions. CFs were seeded into 6-well microplates (Corning, USA) at a density of $1 \times 10^6$/well and cultured in 2 mL of medium. After the treatment as indicated for 48 hours, CFs were continuously incubated with EDU (10 μmol/L) for 2 hours prior to fixation for detection. Proliferating cells were symbolized using bright red fluorescence under a fluorescence microscopy (DP74, Olympus Optical, Tokyo, Japan).

**CCK-8 assay**

Cell proliferation was analyzed by a Cell Counting Kit-8 (CCK8, Beyotime, Shanghai, China) according to the manufacturer's protocols. CFs were seeded into 96-well microplates (Corning, USA) at a density of $5 \times 10^3$/well and cultured in 100 μL of medium. After 48 hours, CFs were incubated with 10 μL of CCK-8 reagent for another 2 hours. The absorbance was analyzed at 450 nm using a microplate reader (Bio-Rad, Hercules, CA, USA), wells without cells as the blanks. The proliferation of cells was expressed by the absorbance.

**Transwell chemotactic assay**

Transwell chemotactic assay was used for measurement of fibroblast migration. Equal numbers of fibroblasts were cultivated on the upper layer of the transwell (Falcon 353097), and culture medium with or without high glucose and palmitic acid were added below the cell permeable membrane. Cells migrated through the membrane were stained with a crystal violet staining solution, and number of cells was counted at 5 random fields under the microscope.

**AAV9 intracardial injection**

The recombinant adeno-associated virus harboring Nr1H2 and scramble sequence were constructed by Hanbio Co., Ltd (Shanghai, China). Mice were anesthetized with 2% isoflurane and fixed to a temperature-controlled panel. Purse sutures were prepared prior to mouse left thoracic skin incision (1.5 cm), and pectoral muscle was dissected directly to expose the ribs. The heart was successfully "ejected" through a muscle incision in the fourth intercostal space. AAV9 was injected into the heart wall with a microliter needle (GA33/15mm /30°, Hamilton, Reno, Nevada) (3 points, 10uL per point, $3 \times 10^{10}$ infection units/ml). The heart was then immediately replaced back, followed by pneumothorax drainage, muscle closure, and surgical sutures.

**Echocardiography**

Echocardiography was carried out as described [2]. Mice were anesthetized by 3%, maintained by 1% isoflurane and fixed on a temperature-controlled panel. Echocardiography was conducted using an echocardiography system with a 15-MHz linear transducer (VisualSonics, Toronto, ON, Canada). At the level of papillary muscles, LV parameters were obtained from the short-axis view using two- dimensional

guided M-mode. Left ventricular ejection fraction (LVEF), left ventricular fraction shortening (LVFS), left ventricular diastolic internal dimension (LVIDd) and left ventricular systolic internal dimension (LVIDs) were calculated by Vevo 2100 software algorithms. All diameters were averaged from 3 consecutive cardiac cycles.

**Chromatin immunoprecipitation (ChIP) assay**

SimpleChIP Plus Sonication Chromatin IP Kit (Cell Signaling Technology) were used to carry out ChIP assay. (1) $5\times10^6$ cells were fixed with 1% methanol for 15 minutes at 37°C. (2) Protease inhibitor cocktail and DTT were added to nucleus extraction buffer and chromatin cutting buffer. (3) Nucleus and chromatin were extracted at 4°C. After chromatin digestion, efficiency of digestion was evaluated. For the best efficiency of digestion, titration was usually required to determine the optimal ratio of micrococcal nuclease to staining quality (4). A protease inhibitor cocktail was added to all buffers and wash solution, and CHIP was performed at 4°C. For a standard chromatin immunoprecipitation, each experimental group (such as samples at different time points treated by the same drug) needs to set up the target protein tube, input sample tube, negative control tube and positive control tube. (5) After addition of antibodies to each of the aforementioned sample tubes, the tube caps were tightly sealed with parafilm and incubated on a rotor at 4°C (can be placed on a turning shaker with a rotation speed of 100 to 150 rpm) overnight. (6) 30 μL of ChIP-grade protein G microbeads (CST # 9006) were added to each of the aforementioned sample tubes and incubated continuously on a rotor at 4 °C for 2 hours. (7) The precipitated chromatin was washed for multiple times with low and high salt washing solutions. (8)Finally, enrichment efficiency analysis and quantitative PCR were used to dectet ChIP DNA after elution and purification of ChIP DNA.

**RhoA activity assay**

Cells were washed three times with PBS, lysed with the kit lysis buffer and protease inhibitor cocktail, and then clarified for 1 min at 10,000 × g at 4°C. The lysate was snap frozen in liquid nitrogen quickly and then stored at −80°C. Protein concentrations were measured using the Bradford reagent. Upon thawing aliquots, lysate protein concentration was normalized to a uniform protein concentration using kit lysis buffer. Equal concentrations of lysate were then passed on 100 µg rhotekin Rho-binding domain beads and incubated at 4°C for 1 h under agitation. The beads were washed, pelleted, and finally boiled with 20 µl Laemmli sample buffer. Samples were run on a 12% split SDS-PAGE gel and blotted for RhoA.

**Western blott**

Proteins were isolated from mouse hearts and CFs. 40μg of each protein sample was separated via 10% or 12% sodium dodecyl sulfate polyacrylamide gel electrophoresis (SDS-PAGE; Cwbiotech, Beijing, China) and transferred onto 0.22 μm nitrocellulose blotting membranes (Millipore, MA, USA). The membranes were blocked with 5% milk for 1 h at 37 °C and incubated with the primary antibodies at 4 °C overnight, and the antigen-antibody complexes were detected by a chemiluminescence system (Amersham Bioscience, Buckinghamshire, UK) after incubation with secondary antibodies for 1 h at 37 °C.

**Quantitative real-time PCR**

Total RNA was extracted using TreliefTM RNAprep FastPure Tissue and Cell Kit (Tsingke, Beijing, China) and reverse transcribed into cDNA using the PrimeScriptRT Reagent Kit (TaKaRa). Then, cDNA was added to the UltraSYBR One Step RT-qPCR Kit (Cwbiotech). All procedures were performed according to the manufacturer's instructions.

**Table 1. Primary antibodies used for flow cytometry, western blotting, immunoprecipitation, and immunohistochemistry**

| Antibody | Working dilutions | Catalog No. | Supplier |
|---|---|---|---|
| Rnd3 | WB: 1/1000<br>IHC: 1/200 | 05-723 | Sigma-Aldrich, MO, USA |
| α-SMA | WB: 1/1000<br>IHC: 1/200 | A17910 | ABclonal, Wuhan, China |
| HES1 | WB: 1/1000 | A0925 | ABclonal, Wuhan, China |
| NICD | WB: 1/1000 | #4147 | CST, MA, USA |
| ROCK1 | WB: 1/1000 | A11158 | ABclonal, Wuhan, China |
| RhoA | WB: 1/1000 | A13947 | ABclonal, Wuhan, China |
| MMP9 | WB: 1/1000 | A0289 | ABclonal, Wuhan, China |
| Collagen I | WB: 1/1000 | A5786 | ABclonal, Wuhan, China |
| TGF-β1 | WB: 1/1000 | ab215715 | Abcam, Cambridge, UK |
| FLAG | WB: 1/1000<br>IP: 1/100 | F7425 | Sigma-Aldrich, MO, USA |
| Ubiquitin | WB: 1/1000 | #3936 | CST, MA, USA |
| Nr1H2 | WB: 1/1000 | A16291 | ABclonal, Wuhan, China |
| GAPDH | WB: 1/50000 | A19056 | ABclonal, Wuhan, China |

**Table 2. Top 10 putative transcription factors of Rnd3**

| Matrix ID | Name | Score | Relative score | Sequence ID | Start | End | Strand | Predicted sequence |
|---|---|---|---|---|---|---|---|---|
| MA1996.1 | MA1996.1.Nr1H2 | 12.495604 | 0.9805551454890508 | NC_000068.8:c51039123-51020451 | 1316 | 1326 | + | CAAAGGTCAAG |
| MA1996.1 | MA1996.1.Nr1H2 | 12.438785 | 0.9792851840183897 | NC_000068.8:c51039123-51020451 | 9 | 19 | + | CAAAGGTCAAT |
| MA1996.1 | MA1996.1.Nr1H2 | 12.006618 | 0.969625973880008 | NC_000068.8:c51039123-51020451 | 493 | 503 | - | GGAAGGTCAGG |
| MA0650.3 | MA0650.3.Hoxa13 | 13.840385 | 0.958120489414654 | NC_000068.8:c51039123-51020451 | 1360 | 1371 | + | TACCAATAAAGC |
| MA1627.1 | MA1627.1.Wt1 | 16.545004 | 0.9453901010116408 | NC_000068.8:c51039123-51020451 | 99 | 112 | + | GCCCTCCCCCACTG |
| MA0650.3 | MA0650.3.Hoxa13 | 12.986913 | 0.9417055689650619 | NC_000068.8:c51039123-51020451 | 1994 | 2005 | - | AACTAATAAAAA |
| MA1125.1 | MA1125.1.ZNF384 | 12.565786 | 0.9341115475547119 | NC_000068.8:c51039123-51020451 | 2589 | 2600 | + | GTACAAAAAAAC |
| MA1125.1 | MA1125.1.ZNF384 | 12.529681 | 0.9335589385950402 | NC_000068.8:c51039123-51020451 | 2613 | 2624 | - | AATTAAAAAACA |
| MA0144.1 | MA0144.1.Stat3 | 13.317728 | 0.9328168012908856 | NC_000068.8:c51039123-51020451 | 598 | 607 | + | TTACAGGAAG |
| MA1627.1 | MA1627.1.Wt1 | 15.679994 | 0.9312548921866468 | NC_000068.8:c51039123-51020451 | 114 | 127 | + | CCCCTCCCCCATGC |

**Table 3. Primer sequences for RT-PCR**

| Mouse | Forward 5' to 3' | Reverse 5' to 3' |
|---|---|---|
| Rnd3 | TTTCGCACATGCCTAGCAGA | CAGATATTCCCGCGTCCTCC |
| Col1a1 | CGATGGATTCCCGTTCGAGT | CAGGAGGGCCATAGCTGAAC |
| α-SMA | CTTCCAGCCATCTTTCATTGG | GTTCTGGAGGGGCAATGAT |
| MMP9 | CGTGTCTGGAGATTCGACTTGA | TGGAAGATGTCGTGTGAGTTCC |
| GAPDH | AGAAGGCTGGGGCTCATTTG | AGGGGCCATCCACAGTCTTC |

**Table 4. Primer sequences for CHIP assay**

| Mouse | Forward 5' to 3' | Reverse 5' to 3' |
|---|---|---|
| Nr1H2 | AACCTAGCCGAGTTTGCAGG | ATGGATTTGGCGGTAGACGG |

**Table 5. The blood glucose and body weight of experimental animals**

| Body weight (g) / Weeks | Control | | | HFD+HTZ | | |
|---|---|---|---|---|---|---|
| | $Cre^{+}$ | $Rnd3^{lsp/lsp}TgCre^{-}$ | $Rnd3^{lsp/lsp}TgCre^{+}$ | $Cre^{+}$ | $Rnd3^{lsp/lsp}TgCre^{-}$ | $Rnd3^{lsp/lsp}TgCre^{+}$ |
| 0 | 21.4±2.1 | 21.8±2.6 | 21.2±1.7 | 21.8±2.6 | 22.4±1.7 | 22.8±1.5 |
| 4 | 22.2±1.6 | 22.4±1.8 | 22.7±1.1 | 24.8±2.0 | 24.4±1.6 | 25.2±1.9 |
| 8 | 23.6±1.1 | 23.1±1.9 | 23.6±1.3 | 27.8±1.9 | 27.2±1.3 | 28.2±1.6 |
| 12 | 24.8±1.3 | 24.6±1.7 | 24.2±1.4 | 28.6±1.9 | 28.2±1.3 | 29.2±1.7 |
| 16 | 26.1±1.4 | 26.9±1.1 | 27.0±1.8 | 33.8±2.3 | 33.0±1.2 | 33.4±1.6 |
| 20 | 28.1±2.0 | 28.6±1.5 | 27.9±2.0 | 38.2±2.2 | 37.8±1.5 | 38.4±1.8 |
| 24 | 29.4±1.9 | 29.6±1.7 | 28.9±2.3 | 42.2±2.6 | 42.8±1.9 | 43.0±1.4 |

| Blood glucose (mmol/L) / Weeks | Control | | | HFD+HTZ | | |
|---|---|---|---|---|---|---|
| | $Cre^{+}$ | $Rnd3^{lsp/lsp}TgCre^{-}$ | $Rnd3^{lsp/lsp}TgCre^{+}$ | $Cre^{+}$ | $Rnd3^{lsp/lsp}TgCre^{-}$ | $Rnd3^{lsp/lsp}TgCre^{+}$ |
| 0 | 7.5±1.2 | 7.8±0.9 | 7.6±1.4 | 7.3±1.1 | 7.9±0.8 | 8.2±1.1 |
| 4 | 7.3±1.4 | 8.1±0.9 | 8.0±1.1 | 7.5±0.6 | 6.7±1.0 | 6.9±0.9 |
| 8 | 7.2±1.3 | 7.9±1.0 | 7.8±0.9 | 7.2±1.0 | 7.0±1.4 | 6.5±1.2 |
| 12 | 19.9±1.9 | 20.5±1.9 | 19.6±2.3 | 7.3±1.3 | 7.1±1.7 | 7.4±1.6 |
| 16 | 24.5±1.7 | 26.4±1.5 | 25.3±2.0 | 6.9±1.2 | 6.4±1.4 | 7.6±1.5 |
| 20 | 22.8±1.8 | 23.1±2.1 | 23.9±1.8 | 7.0±1.4 | 6.7±1.2 | 7.3±1.2 |
| 24 | 20.5±1.6 | 21.3±1.8 | 21.9±1.6 | 6.4±1.4 | 7.4±1.3 | 6.9±1.4 |

| Body weight (g) / Weeks | Control | | | HFD+HTZ | | |
|---|---|---|---|---|---|---|
| | $Cre^{+}$ | $Rnd3^{fl/fl}KOCre^{-}$ | $Rnd3^{fl/fl}KOCre^{+}$ | $Cre^{+}$ | $Rnd3^{fl/fl}KOCre^{-}$ | $Rnd3^{fl/fl}KOCre^{+}$ |
| 0 | 21.6±1.9 | 21.6±2.6 | 20.8±1.5 | 22.2±1.5 | 21.4±1.1 | 21.6±1.3 |
| 4 | 22.7±1.1 | 22.5±1.8 | 22.2±1.6 | 25.0±1.2 | 24.6±1.2 | 24.2±1.3 |
| 8 | 23.5±1.3 | 23.8±1.3 | 22.9±1.7 | 27.6±1.7 | 27.2±0.8 | 28.2±1.8 |
| 12 | 24.3±1.5 | 25.1±1.4 | 24.3±1.4 | 28.8±1.9 | 28.0±1.4 | 29.2±1.6 |
| 16 | 26.7±1.9 | 26.6±1.4 | 26.1±0.9 | 34.2±1.8 | 32.8±1.9 | 33.2±1.3 |
| 20 | 27.8±1.9 | 27.9±2.1 | 28.0±1.2 | 38.6±1.5 | 37.6±2.3 | 38.2±1.3 |
| 24 | 28.9±2.3 | 28.6±1.8 | 29.6±1.2 | 42.3±1.5 | 42.2±2.6 | 43.2±1.8 |

| Blood glucose (mmol/L) / Weeks | Control | | | HFD+HTZ | | |
|---|---|---|---|---|---|---|
| | $Cre^{+}$ | $Rnd3^{fl/fl}KOCre^{-}$ | $Rnd3^{fl/fl}KOCre^{+}$ | $Cre^{+}$ | $Rnd3^{fl/fl}KOCre^{-}$ | $Rnd3^{fl/fl}KOCre^{+}$ |
| 0 | 8.1±1.0 | 7.7±1.4 | 7.2±1.0 | 7.9±0.8 | 6.9±1.3 | 6.9±1.4 |
| 4 | 7.3±1.4 | 7.6±1.2 | 7.8±0.7 | 7.5±0.6 | 6.7±1.0 | 7.1±1.0 |
| 8 | 7.7±1.4 | 8.0±1.0 | 7.9±0.8 | 7.2±1.0 | 6.9±1.4 | 6.9±1.3 |
| 12 | 19.7±0.9 | 20.7±1.0 | 20.4±1.8 | 7.6±1.1 | 6.8±1.8 | 7.7±1.6 |
| 16 | 24.4±2.3 | 25.9±1.5 | 25.4±1.8 | 6.7±1.2 | 6.4±1.0 | 7.4±1.3 |
| 20 | 23.8±1.8 | 23.2±1.7 | 23.0±1.1 | 7.5±1.6 | 6.3±0.8 | 7.0±1.2 |
| 24 | 20.9±1.7 | 21.2±1.0 | 20.8±1.2 | 6.8±1.4 | 7.1±1.6 | 7.3±1.8 |

# Supplemental Figures and Figure Legends

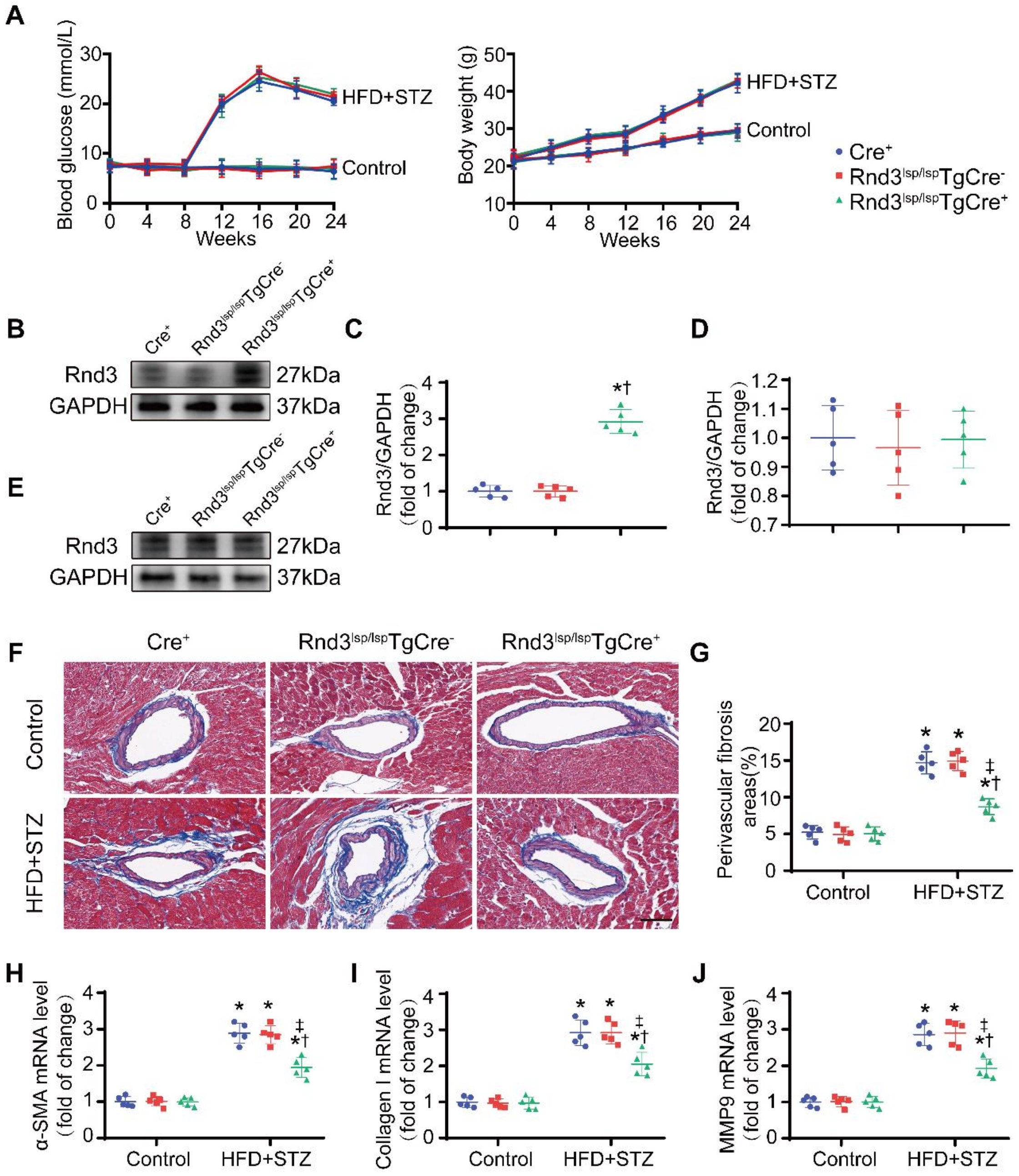


**Figure S1. Evaluation of cardiac fibrosis in mice overexpressing Rnd3.** (A) Blood glucose and body weight were examined at 0, 4, 8, 12, 16, 20, 24 weeks after the indicated treatments in $Cre^{+}$ mice, $Rnd3^{lsp/lsp}TgCre^{-}$ mice, and $Rnd3^{lsp/lsp}TgCre^{+}$ mice (n = 8). (B-C) Protein level of Rnd3 was evaluated in cardiac fibroblasts from $Cre^{+}$, $Rnd3^{lsp/lsp}TgCre^{-}$, and $Rnd3^{lsp/lsp}TgCre^{+}$ mice for validation of mouse models (n = 5). (D-E) Protein level of Rnd3 was evaluated in cardiomyocytes of $Cre^{+}$ mice, $Rnd3^{lsp/lsp}TgCre^{-}$ mice, and $Rnd3^{lsp/lsp}TgCre^{+}$ mice to verify mouse models (n = 5). *$P < 0.05$ *vs.* $Cre^{+}$; †$P < 0.05$ *vs.* $Rnd3^{lsp/lsp}TgCre^{-}$. (F-G) Masson's trichrome staining was used to evaluate perivascular fibrosis in mouse hearts; scale bars

represent 50 μm (n = 5). (H) qPCR analysis of α-SMA expression (n = 5). (I) qPCR analysis of Collagen I expression (n = 5). (J) qPCR analysis of MMP9 expression (n = 5). *P < 0.05 *vs.* control; †P < 0.05 *vs.* HFD+STZ-Rnd3$^{lsp/lsp}$TgCre$^-$; ‡P < 0.05 *vs.* HFD+STZ-Rnd3$^{lsp/lsp}$TgCre$^+$.

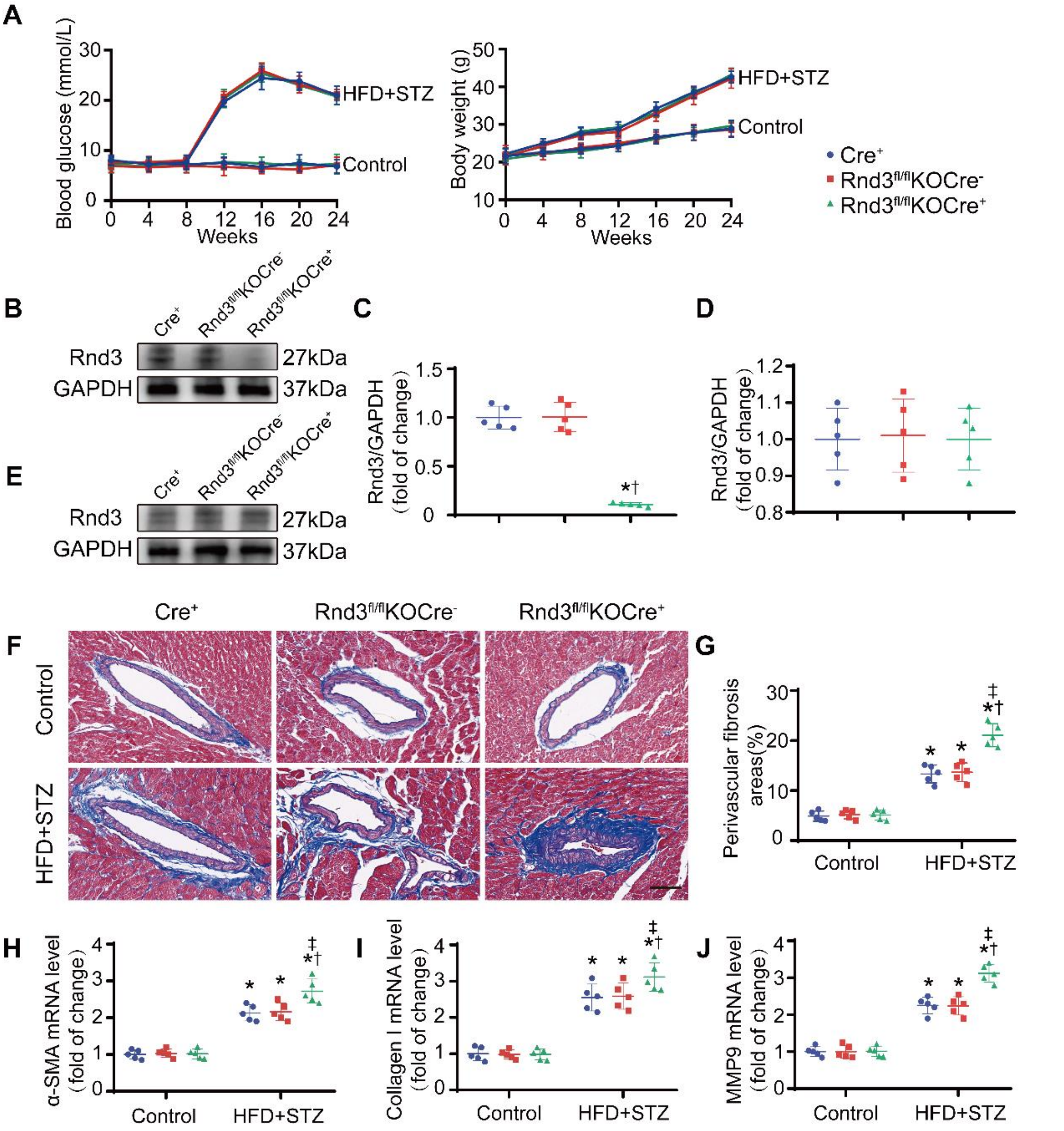


**Figure S2. Evaluation of cardiac fibrosis in mice with Rnd3 knockout.** (A) Blood glucose and body weight were examined at 0, 4, 8, 12, 16, 20, 24 weeks following indicated treatments in Cre$^+$ mice, Rnd3$^{fl/fl}$KOCre$^-$ mice, and Rnd3$^{fl/fl}$KOCre$^+$ mice (n = 8). (B-C) Protein level of Rnd3 was evaluated in cardiac fibroblasts of Cre$^+$, Rnd3$^{fl/fl}$KOCre$^-$, and Rnd3$^{fl/fl}$KOCre$^+$ mice to validate mouse models (n = 5). (D-E) Protein level of Rnd3 was evaluated in cardiomyocytes of Cre$^+$ mice, Rnd3$^{fl/fl}$KOCre$^-$ mice, and Rnd3$^{fl/fl}$KOCre$^+$ mice to validate mouse models (n = 5). *P < 0.05 *vs.* Cre$^+$; †P < 0.05 *vs.* Rnd3$^{fl/fl}$KOCre$^-$. (F-G) Masson's trichrome staining was used to evaluate perivascular fibrosis in mouse hearts; scale bars represent 50 μm (n = 5). (H) qPCR analysis of α-SMA expression in different treatment groups (n = 5). (I) qPCR analysis of Collagen I expression (n = 5). (J) qPCR analysis of MMP9 expression (n = 5). *P < 0.05 *vs.* control; †P < 0.05 *vs.* HFD+STZ-Rnd3$^{fl/fl}$KOCre$^-$; ‡P < 0.05 *vs.* HFD+STZ-Rnd3$^{fl/fl}$KOCre$^+$.

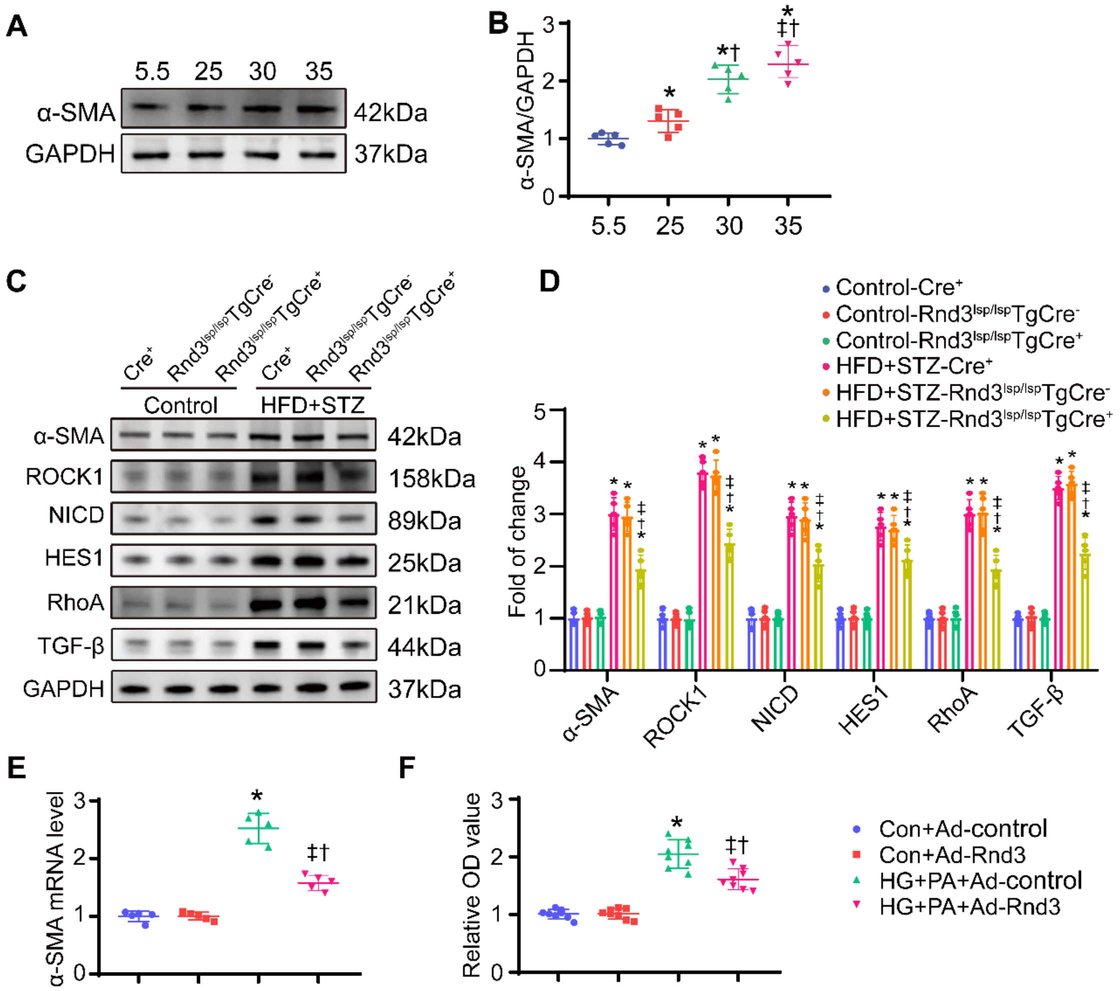


**Figure S3. Evaluation of activation status and related signaling changes in fibroblast.** (A-B) The evaluation and quantitative analysis of fibroblast activation status under different levels of glucose in CFs treated as indicated (n = 5). *P < 0.05 *vs.* 5.5mM; †P < 0.05 *vs.* 25mM; ‡P < 0.05 *vs.* 30mM. (C-D) Activation and related signaling changes in fibroblast isolated from adult mice (n = 5). *P < 0.05 *vs.* control; †P < 0.05 *vs.* HFD+STZ-$Rnd3^{lsp/lsp}TgCre^{-}$; ‡P < 0.05 *vs.* HFD+STZ-$Rnd3^{lsp/lsp}TgCre^{+}$. (E) qPCR analysis of α-SMA expression in various treatment groups (n = 5). (F) Cells proliferation assay of cardiac fibroblast using CCK-8 assay (n = 8). *P < 0.05 *vs.* Con+Ad-control; †P < 0.05 *vs.* con+Ad-Rnd3; ‡P < 0.05 *vs.* HG+PA+Ad-control.

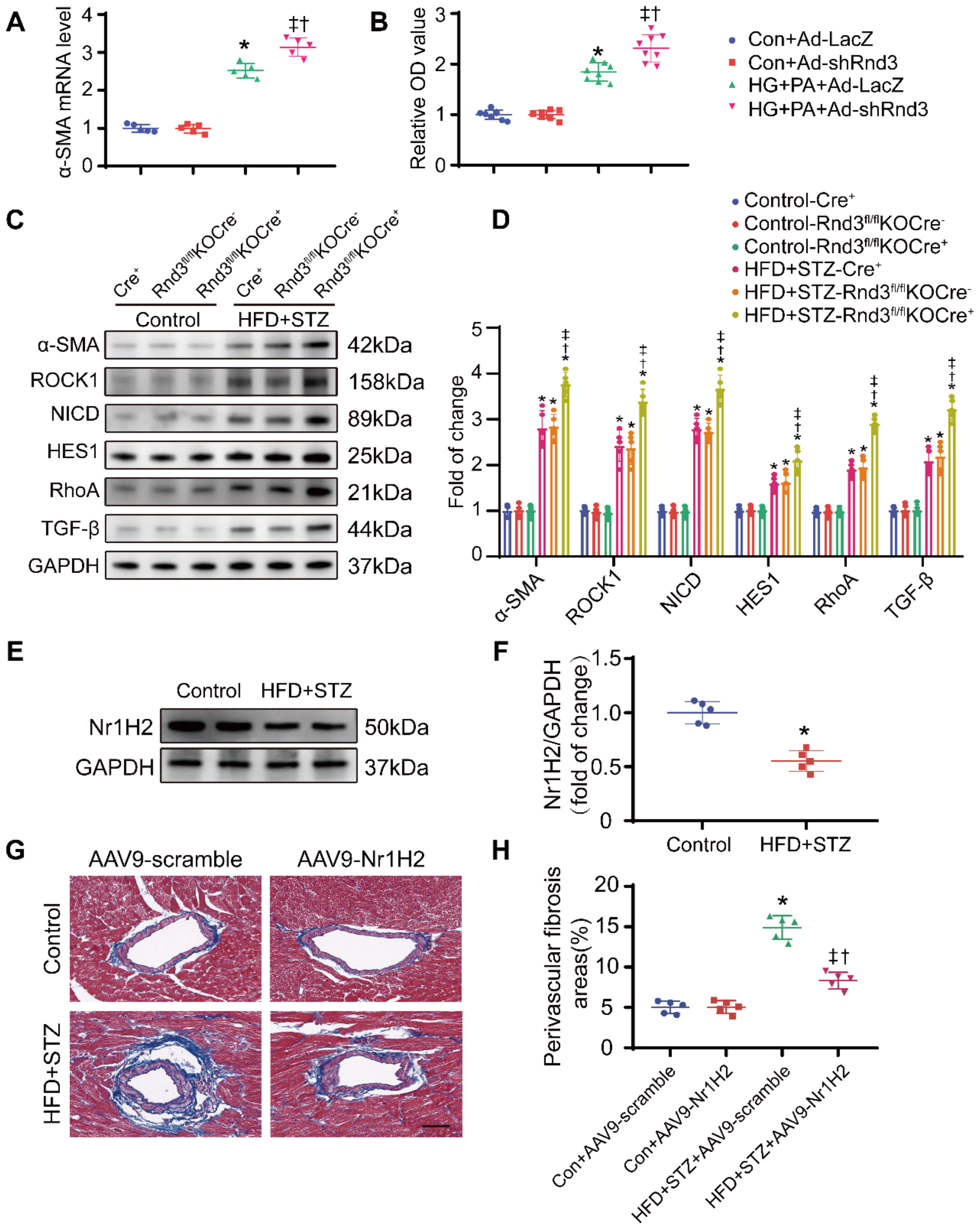


**Figure S4. Evaluation of activation and related signaling changes in fibroblasts.** (A) qPCR analysis of α-SMA expression in various treatment groups (n = 5). (B) Cells proliferation assay of cardiac fibroblast using CCK-8 assay (n = 8). *P < 0.05 *vs.* Con+Ad-LacZ; †P < 0.05 *vs.* con+Ad-shRnd3; ‡P < 0.05 *vs.* HG+PA+Ad-LacZ. (C-D) The activation status and related signaling changes in fibroblast isolated from adult mice (n = 5). *P < 0.05 *vs.* control; †P < 0.05 *vs.* HFD+STZ-Rnd3$^{fl/fl}$KOCre$^-$; ‡P < 0.05 *vs.* HFD+STZ-Rnd3 $^{fl/fl}$KOCre$^+$. (E-F) Representative and associated quantitative analysis of Nr1H2 in cardiac treated as indicated (n = 5). *P < 0.05 *vs.* control. (G-H) Masson's trichrome staining was used to evaluate perivascular fibrosis in mouse hearts; scale bars represent 50 μm (n = 5). *P < 0.05 *vs.* Con+AAV9-scramble; †P < 0.05 *vs.* con+AAV9-Nr1H2; ‡P < 0.05 *vs.* HFD+STZ+AAV9-scramble.
